# Empirical model to assess leaching of pesticides in soil under a steady-state flow and tropical conditions

**DOI:** 10.1007/s13762-023-05038-w

**Published:** 2023-08-20

**Authors:** C. S. Mosquera-Vivas, R. E. Celis-Ossa, C. A. González-Murillo, N. Obregón-Neira, M. J. Martínez-Cordón, J. A. Guerrero-Dallos, G. García-Santos

**Affiliations:** 1 Departamento de Química, Facultad de Ciencias, Universidad Nacional de Colombia, Avenue 45th, 111321 Bogotá, D.C., Colombia; 2 Departamento de Ingeniería Civil y Agrícola, Facultad de Ingeniería Civil, Universidad Nacional de Colombia, Avenue 45th, 111321 Bogotá, D.C., Colombia; 3 Departamento de Ingeniería Civil, Pontificia Universidad Javeriana, Avenue 7th, 110231 Bogotá, D.C., Colombia; 4 Department of Geography and Regional Studies, Alpen-Adria-University, Lakesidepark Haus B02, Ebene 2, 9020 Klagenfurt, Austria; 5 Departamento de Química, Facultad de Ciencias, At current Departamento de Agronomía, Facultad de Ciencias Agrarias, Universidad Nacional de Colombia, Avenue 45th, 111321 Bogotá, D.C., Colombia

**Keywords:** Pollutant, Transport, Adsorption–desorption, Degradation, Simulation

## Abstract

**Abstract:**

An empirical model of leaching of pesticides was developed to simulate the concentration of fungicides throughout unsaturated soil. The model was based on chemical reactions and the travel time of a conservative tracer to represent the travel time required for water to flow between soil layers. The model’s performance was then tested using experimental data from dimethomorph and pyrimethanil applied to the soil under field and laboratory conditions. The empirical model simulated fungicide concentration on soil solids and in soil solution at different depths over time (mean square error between 2.9 mg^2^ kg^−2^ and 61mg^2^ kg^−2^) using sorption percentages and degradation rates under laboratory conditions. The sorption process was affected by the organic carbon, clay, and the effective cation exchange capacity of the soil. The degradation rate values of dimethomorph (0.039 d^−1^–0.009 d^−1^) and pyrimethanil (0.053 d^−1^–0.004 d^−1^) decreased from 0 to 40 cm and then remained constant in deeper soil layers (60–80 cm). Fungicide degradation was a critical input in the model at subsurface layers. The model was determined to be a reliable mathematical tool to estimate the leachability of pesticides in tropical soil under a steady-state flow. It may be extended to other substances and soils for environmental risk assessment projects.

**Graphical abstract:**

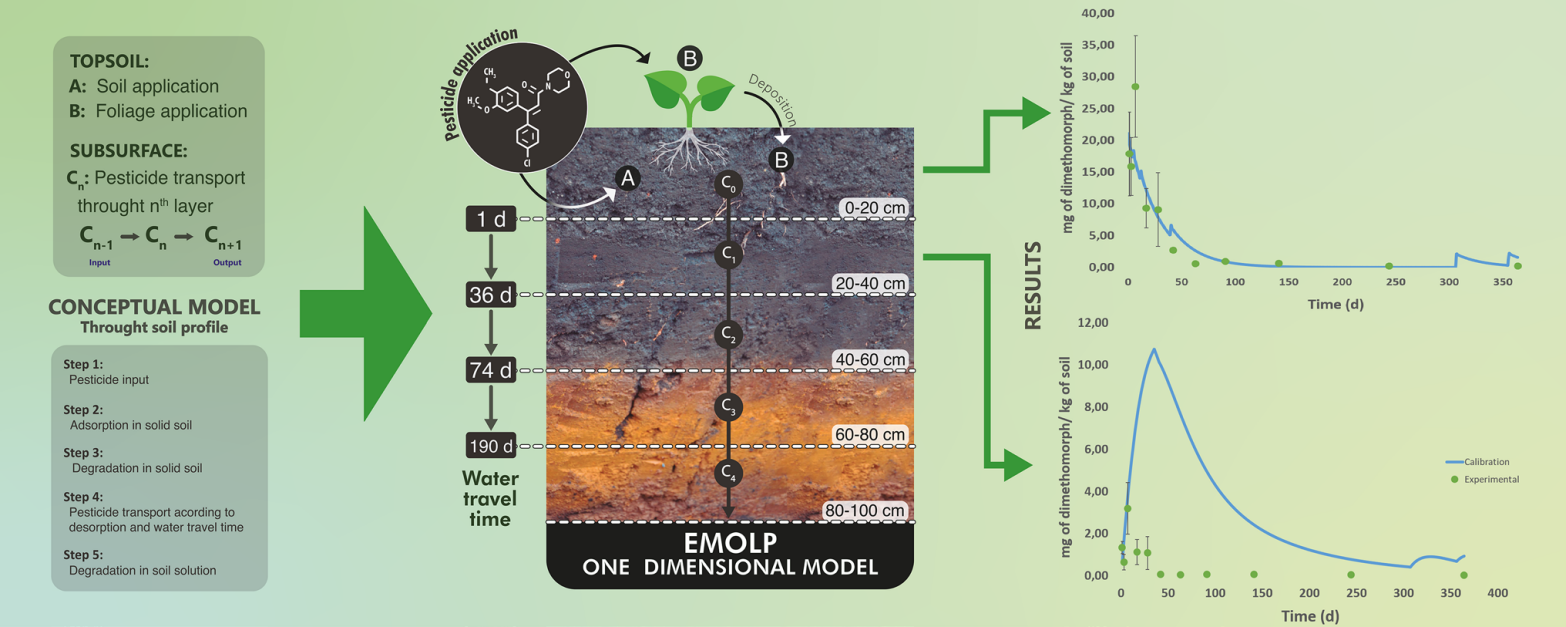

**Supplementary Information:**

The online version contains supplementary material available at 10.1007/s13762-023-05038-w.

## Introduction

The moderate exponential growth followed by the accelerated explosive increase of the human population (Keinan and Clark 2012) has led to high demand for food, fibers, commodities, and raw materials. Pesticides play an important role in agricultural production, but their long-term use has negative effects on living organisms (Dhuldhaj et al. 2023; Pathak et al 2022), pest resistance (Ma et al. 2021), and pollution of the soil (Fang et al. 2017; Saha et al. 2021), water (Gaona et al. 2019; Oltramare et al. 2023), and air (Zaller et al. 2022; Srimurali et al. 2015).

The soil plays a fundamental role in the environmental fate of pesticides (Juraske et al. 2011; Mosquera-Vivas et al. 2016a; Ramakrishnan et al. 2019). Once pesticides reach the soil, they can be taken up by plants (Jorda et al. 2021), carried away by surface runoff (Didoné et al. 2021), degraded by microorganisms (Bragança et al. 2019; Fenoll et al. 2011; Piao et al. 2011), adsorbed or desorbed from soil solids (Kaur et al. 2023; Mosquera-Vivas et al. 2018; Vanni et al. 2006), and leached through the soil profile to groundwater (Park et al. 2020).

The assessment of pesticide leaching in tropical soils has been mainly based on: (i) environmental monitoring of soil, sediments, and water samples (de Azeredo Morgado et al. 2023; Zhou et al. 2023); (ii) the use of compound ranking indexes such as the groundwater ubiquity index (GUS), the retardation factor (RF), the attenuation factor (AF), the log-transformed attenuation factor (AFT), and the Comprehensive Leaching Risk Assessment System (CLEARS) (Bernard et al. 2005; Hall et al. 2015; Mosquera-Vivas et al. 2018); (iii) column experiments (Laabs et al. 2002); and (iv) pesticide simulation models, including PESTicide Fate And Dynamics in the Environment (PESTFADE), the Root Zone Water Quality Model (RZWQM), the Soil & Water Assessment Tool (SWAT), and the WISORCH and WATPPASS fate models (Bannwarth et al. 2014; Cabidoche et al. 2009; Mottes et al. 2015; Shrestha and Datta 2015). Pesticide fate models require information about climate, agricultural practices, physicochemical properties of both the pesticide and the soil, pesticide interception by the crop, pesticide in runoff, soil hydraulic parameters, and water balance. Climate variability, chemical reaction of the substances (adsorption and half-life of pesticides), soil hydraulic conductivity, and spatial variability in rainfall are the most influential factors in pesticide-leaching fluxes through the soil profile (Heuvelink et al. 2010; Lammoglia et al. 2019). Undisturbed or disturbed soil columns are essential for estimating model parameters and testing pesticide fate models and screening indexes (Aslam et al. 2018; Haddad et al. 2019; Khan and Brown 2016; Laabs et al. 2002). Numerous models have been developed to simulate pesticide fate in soils. In these models, the runoff is simulated with the Soil Conservative Service Curve Numbers and Modified Universal Soil Loss Equation, the heat flow with the second law of heat conduction, the water flow with Richards’ equation and the percolation rate, and the solute transport with the advection–dispersion equation and the soil pesticide transfer component. Furthermore, the models use the RF for instantaneous and reversible sorption, the first-order desorption kinetics for long-term pollution, and Bayesian methods for calibration (Aslam et al. 2018; Cabidoche et al. 2009; McGrath et al. 2019; Šimůnek and van Genuchten 2008). These tools are all very useful in the hands of experts with high-quality data and computational packages. However, farmers and environmental agencies need simple but reliable models based on a minimum of processes for simulating pesticide concentration throughout the soil profile. There is thus a need for models that are accurate but that require less data under situations where information on the soil and pesticide conditions is limited, as frequently occurs in countries in the tropics. Parsimonious models could be implemented using computational systems that require fewer technical resources, such as mobile applications designed for use in the field.

The chemical reaction of the compounds of concern is a critical input in most pesticide leaching models for assessing their likelihood of occurrence in water sources (Demir et al. 2019; Heuvelink et al. 2010; Khan and Brown 2016). Adsorption–desorption assays characterize the mobility of a substance in the soil, and the degradation process is important for calculating the persistence and off-site impact of pesticides. These processes have been widely studied for the fungicides dimethomorph and pyrimethanil in temperate environments in the upper soil layer (FAO 2014a, b; Fenoll et al. 2010; Langeron et al. 2014; PPDB 2022; Vanni et al. 2006; Wang et al. 2017) and some subsurface soil layers (Capri et al. 2001). For instance, dimethomorph showed values of the Freundlich adsorption affinity ($K_{{{\text{fa}}}}$) between 2.1 and 19.7, Freundlich organic carbon-normalized adsorption affinity ($K_{{{\text{foc}}}}$) between 290 and 566, and half-lives ($t_{{\left( {1/2} \right)}}$) between 16 and 599 d in laboratory assays and between 1.9 d and 61 d under field conditions (FAO 2014a; Liang et al. 2011b; Liu et al. 2012; Piao et al. 2011; PPDB 2022; Wang et al. 2017; Zhang et al 2022). Pyrimethanil presented the following sorption, degradation, and dissipation values: $K_{{{\text{fa}}}}$: 1.2–273.5, $K_{{\text{d}}}$: 3.9–18.2 L kg^−1^, $K_{{{\text{oc}}}}$: 177–607 L kg^−1^, $t_{{\left( {1/2} \right)}}$ between 23 d and 693 d in laboratory trials and 5.3 d–34.5 d under field conditions (Capri et al. 2001; FAO 2014b; Fenoll et al. 2011; Langeron et al. 2014; Mukherjee et al. 2016; PPDB 2022; Vanni et al. 2006; Yu et al. 2010). Increasing soil organic matter (SOM) and clay content increased the retention of pyrimethanil (Capri et al. 2001; Langeron et al. 2014; Yu et al. 2010).

Despite this extensive body of work in temperate systems, the chemical reactions of the fungicides dimethomorph and pyrimethanil throughout the soil profile in tropical regions are still poorly understood. Therefore, more studies are needed. This is important because some pesticide research has shown that adsorption and degradation processes differ between temperate and tropical soils. For instance, the organic carbon-normalized adsorption coefficient of some pesticides has been reported to differ by an order of magnitude between tropical and temperate conditions (Ahmad et al. 2001). The dissipation of chlorpyrifos, dimethomorph, and pyrimethanil in the surface soil layer apparently occurred more rapidly under tropical conditions (Mosquera-Vivas et al. 2017; Ngan et al. 2005; Redondo et al. 1997), while the decrease of SOM through the tropical soil profile decreases chlorpyrifos degradation and increases the risk of insecticide pollution of groundwater (Mosquera-Vivas et al. 2016a). Therefore, the calibration of pesticide leaching models requires local parameters derived from tropical environments; simply using pesticide chemical parameters obtained from temperate topsoil could lead to overestimation or underestimation of pesticide concentration throughout the soil profile.

The aims of this study are therefore to (i) assess the chemical reaction (sorption and degradation) of the fungicides dimethomorph and pyrimethanil through the soil profile under laboratory conditions; (ii) develop a new and reliable mathematical tool, here called Empirical Model to assess Leaching of Pesticides (EMOLP), to estimate the leaching of fungicides in the soil profile via chemical reaction; and (iii) to calibrate, validate, and evaluate the EMOLP (“tipping bucket type model”) under field conditions at soil depths between 0 and 100 cm. The study was carried out in Tenjo (Cundinamarca, Colombia) between 2012 and 2014. EMOLP is a novel pesticide leaching model with new mathematical equations developed for tropical soils under steady-state flow conditions. The model facilitates the estimation of changes in pesticide concentration over time in the solid and aqueous phases at different depths, which contributes to a better understanding of the movement of pesticides in the tropical soils. Furthermore, the EMOLP model can be used in other soils as an environmental assessment tool for the lithosphere, the hydrosphere, the biosphere, and groundwater quality sensing.

## Materials and methods

### Study area

The field trials were carried out in a 6587 m^2^ rose crop greenhouse located in the municipality of Tenjo (Cundinamarca, Colombia) with coordinates 4°50′31.5″N/74°10′18.3″W, at 2595 m above sea level. The greenhouse soil was Thaptic Hapludands (Mosquera-Vivas et al. 2016a). The soil was under a steady flow and the pressure at a depth of 15 cm was − 30 kPa (0.42 ± 0.04 m^3^/m^3^ with n = 78). The main soil properties are summarized in Table 1 (see also Mosquera-Vivas et al. (2016a) for further details). The rose crop was cultivated at a slope between 0 and 3% in furrows measuring 31 m × 0.8 m, irrigated daily using trickle irrigation (between 2.4 and 3.0 mm d^−1^), and the crop stage varied during the trials.

**Table 1:** Physical and chemical soil properties for the Andisol soil profile

Soil type	Layer (depth)	pH^a^	pH classification	OC^b^, %	CEC^c^, cmol( +) kg^−1^	ECEC^d^, cmol( +) kg^−1^	Clay^e^, %	Silt^e^, %	Sand^e^, %	Textural classification
Andisol	SL1 (0–20 cm)	6.3 ± 0.2	Slightly acid	10.3 ± 0.3	57.8	49.1	16.0	20.0	52.0	Loam
	SL2 (20–40 cm)	5.8 ± 0.1	Moderately acid	10.7 ± 0.8	58.0	34.0	16.0	32.0	52.0	Loam
	SL3 (40–60 cm)	5.2 ± 0.3	Strongly acid	9.6 ± 0.4	46.5	17.6	14.0	20.0	66.0	Sandy loam
	SL4 (60–80 cm)	5.3 ± 0.2	Strongly acid	4.8 ± 0.7	38.2	9.0	10.0	12.0	78.0	Sandy loam
	SL5 (80–100 cm)	5.4 ± 0.1	Strongly acid	2.7 ± 0.8	32.6	7.2	6.0	18.0	76.0	Sandy loam

^*a*^ 1:1 water-soil ratio; ^*b*^ Walkley–Black method; ^*c*^ CEC (cationic exchange capacity) is obtained by ammonium at pH = 7.0 and exchange with NaCl with units of cmol( +) kg^−1^ g of dry soil; ^*d*^ ECEC (effective cationic exchange capacity) is the sum of the ions extracted with ammonium at pH = 7.0 with units of cmol( +) kg^−1^ g of dry soil, ^*e*^ Bouyoucos method. These properties were acquired in Mosquera-Vivas et al. (2016a)

The ± sing is the standard deviation of mean

### Pesticides

The fungicides selected for the laboratory and field trials were dimethomorph [(EZ)-4-[3-(4-chlorophenyl)-3-(3,4-dimethoxyphenyl)acryloyl]morpholine] and pyrimethanil [N-(4,6-dimethylpyrimidin-2-yl)aniline]. According to a multicriterion analysis, these pesticides had the highest probability of detection in groundwater samples among 27 frequently used active ingredients (Table S3 in Supporting Information). Dimethomorph is used to control downy mildew and is applied directly to the soil and leaves, and pyrimethanil is sprayed on the flower bud to control botrytis in cut rose crops. The fungicides (≥ 98% purity) were provided by Dr. Ehrenstorfer GmbH (Augsburg, Germany). Forum ® (500 g L^−1^ dimethomorph) and Scala ® (408 g L^−1^ pyrimethanil) were acquired from BASF (Ludwigshafen am Rhein, Germany) and Bayer Crop-Science (Monheim am Rhein, Germany), respectively.

### Chemical reaction of the fungicides under laboratory conditions

Adsorption, desorption, and degradation processes allow the description of the fate of pesticides in the vadose zone, such as the likelihood of being leached into the groundwater (Huang et al. 2019). The adsorption and the single-point desorption isotherms of the pesticides in soils were investigated using the batch equilibrium method (Cueff et al. 2020; Dotor-Robayo et al. 2022; Gámiz et al. 2019; López-Piñeiro et al. 2022). The single-point desorption isotherm is the plot of the amount of pesticide adsorbed versus the compound concentration in the supernatant solution after the first 24 h of desorption (Mamy and Barriuso 2007), and it represents a single point in the space or non-spatialized model of the plane. We used this kind of isotherm as a mathematical description of the relationship between the amount of adsorbed fungicide and its concentration in solution after 24 h of desorption (Mamy and Barriuso 2007).

The fungicides dimethomorph and pyrimethanil in Andisol (soil layers, SL1: 0–20 cm, SL2: 20–40 cm, SL3: 40–60 cm, SL4: 60–80 cm, SL5: 80–100 cm) were studied together in a mixture solution. The five initial concentrations of dimethomorph and pyrimethanil varied from 0.3 mg L^−1^ to 5.4 mg L^−1^ and from 0.6 mg L^−1^ to 9.8 mg L^−1^ in 0.01 M CaCl_2_, respectively. The range of the initial fungicide concentrations was based on the typical field doses of dimethomorph (0.64 kg a.i. ha^−1^) and pyrimethanil (1.06 kg a.i. ha^−1^), assuming a homogeneous distribution in the first 20 cm of the soil profile. The concentrations ranged from one-half to seven times the field doses and were calculated using the soil bulk density (SL1 = 0.48 kg L^−1^) and the field water condition (SL1 = 88.10 g/100 g) of the topsoil. The range of initial concentrations of the fungicides in the crop included a frequently applied overdose (as observed by Mojica and Guerrero (2013)) and was used to test the adsorption energies as a function of the adsorbate concentration (Mosquera-Vivas et al. 2018). The overdose concentration represented the worst-case scenario in the study of the fate of pesticides in the soil.

Ten (10.00) grams of dry soil per duplicate (SL1-SL5) was placed in 50-mL centrifuge tubes, and a 20.0 mL aliquot of the pesticide mixture was added to each tube. The soil–water suspension was shaken for 24 h at 20 °C and centrifuged at 7000 r min^−1^ for 30 min. The supernatant was removed, weighed, extracted by liquid–liquid extraction with ethyl acetate, and concentrated to a volume of 2 mL (Mosquera-Vivas et al. 2016b). The single-point desorption for each sorption amount was obtained using the same soil layers as the adsorption process. After the adsorption, the supernatant was replaced with 20.0 mL of 0.01 M CaCl_2_ without fungicides. The soil–water slurry was shaken for 24 h at 20 °C and centrifuged at 7000 r min^−1^ for 30 min. The supernatant was removed, weighed, and extracted by liquid–liquid extraction as above. The volume of solution retained in the soil after the adsorption assay was included in the calculations. According to Huang et al. (1998), the use of a single-desorption measurement technique assumes that both the adsorption and desorption isotherms for a given system are linear. Furthermore, apparent desorption hysteresis determined from single-step observation is three times as large as the value reported using the Freundlich model.

All of the final organic extracts were injected into an Agilent Technologies (Santa Clara, CA) Model 7890A Gas Chromatograph coupled with a 5975C Mass Selective Detector, GC-MSD. The expected recoveries of the fungicides with this method were 85.5 ± 7.1% for dimethomorph and 84.5 ± 6.2% for pyrimethanil. The limits of detection (LOD) of the pesticides were estimated to be 0.60 μg mL^−1^ for dimethomorph and 0.24 μg mL^−1^ for pyrimethanil, and the limits of quantification (LOQ) were estimated as 2.00 μg mL^−1^, 0.78 μg mL^−1^ for dimethomorph and pyrimethanil, respectively. Details of the pesticide quantification are given in Mosquera-Vivas et al. (2016b).

Adsorption and single-point desorption data were fitted to the Freundlich and linear models:

1 $$ x/m = K_{y} *C_{{{\text{eq}}}}^{n} $$

where $x/m$ is the amount of pesticide adsorbed per unit mass (mg kg^−1^), $K_{y}$ is the coefficient of distribution (L kg^−1^) or the adsorption affinity of pesticide [(mg kg^−1^) (mg L^−1^)] ^*n*^, $C_{{{\text{eq}}}}$ is the equilibrium concentration of the pesticide in the soil solution (mg L^−1^), and $n$ is the energy of adsorption. When $n$ was equal to 1, the Freundlich model was simplified to a linear equation. In addition, the retention data were used to calculate the adsorption and desorption percentages as:

2 $$ \% {\text{ads}} = {\raise0.7ex\hbox{${{\text{adsorbed}}\;{\text{pesticide}} \left( {{\text{mg}}} \right)}$} \!\mathord{\left/ {\vphantom {{{\text{adsorbed}}\;{\text{pesticide}} \left( {{\text{mg}}} \right)} {{\text{added}}\;{\text{pesticide }}\left( {{\text{mg}}} \right)}}}\right.\kern-0pt} \!\lower0.7ex\hbox{${{\text{added}}\;{\text{pesticide }}\left( {{\text{mg}}} \right)}$}}* 100\% $$

3 $$ \% {\text{des}} = \% {\text{ads}} - \% {\text{ads}}_{24} $$

where $\%ads$ is the percentage of adsorption, $\%des$ is the percentage of desorption, and ${\%ads}_{24}$ is the percentage of adsorption after the single-point desorption.

To understand the process of pesticide adsorption onto soils, we estimated the partial molar free energy ${\Delta G}_{ads}^{\circ }$ and the enthalpy ${\Delta H}_{ads}$ of adsorption using a thermodynamic approach based on the equations proposed by Mosquera-Vivas et al. (2016b); Singh and Srivastava (2009). In addition, the effect of soil pH on pesticide adsorption was estimated using the protonated and deprotonated species concentrations. All of the equations are included in Supporting Information (Eq. (S2), Eq. (S3), Eq. (S4), Eq. (S5), and Eq. (S6)).

The degradation rates ($k$) of the fungicides in Andisol were acquired using the laboratory incubation method and the first-order kinetic model (Mosquera-Vivas et al. 2016b). Dimethomorph (12.1 kg a.i. ha^−1^) and pyrimethanil (20.2 kg a.i. ha^−1^) were added to 10.0 g of dry soil (SL1-SL5) with field water capacity between 0.40 m^3^ m^−3^ and 0.46 m^3^ m^−3^. After each incubation time (0, 1, 3, 7, 14, 31, 45, 60, 76, and 102 days), two soil samples from each depth were dried, extracted with ethyl acetate (20 mL), centrifuged at 7000 r min^−1^, and concentrated. The final organic extracts were injected into the GC–MS system. The LOD of the pesticides was estimated to be 0.040 μg g^−1^ for dimethomorph and 0.036 μg g^−1^ for pyrimethanil, and the LOQ was 0.135 μg g^−1^ for dimethomorph and 0.071 μg g^−1^ for pyrimethanil. The recovery of the fungicides ranged from 83.5% to 97.9%.

### Empirical model to assess leaching of pesticides, EMOLP

The EMOLP was developed for the vadose zone at a depth of 0–100 cm. It was based on: (i) adsorption–desorption equilibrium, (ii) a sink of the pesticides in solid and liquid phases of the soil using the first-order kinetic model, and iii) the travel time required for water flow, which was estimated based on the travel time of a conservative tracer in an undisturbed column assay. The soil profile (0–100 cm) was divided into five soil layers with a thickness of 20 cm each (Fig. S1 in Supporting Information). A description of the calibration and validation of the EMOLP model, i.e., input and output concentrations of fungicides and intermediate processes like adsorption, desorption, degradation, and transport in the soil layers (topsoil and subsurface), is described in the following steps:

#### Topsoil layer (0–20 cm)

*Step 1*: The pesticide concentration was applied to the soil and/or leaves. *Step 2*: The amount of pesticide adsorbed onto soil solids was estimated according to the adsorption percentage, while the pesticide that was not adsorbed remained in the soil solution. This accounts for instantaneous adsorption. We used this method because the adsorption percentages were very similar among different initial concentration ranges of dimethomorph (0.3–5.4 mg L^−1^ and 0.3–1.6 mg L^−1^) and pyrimethanil (0.6–9.8 mg L^−1^ and 0.6–2.1 mg L^−1^), while the ${K}_{d}$ values of the fungicides varied with the initial concentration range (Table S4 in Supporting Information). *Step 3*: The pesticide adsorbed onto soil solids was assumed to degrade with a first-order kinetic model. Although Ogram et al. (1984) showed that sorbed pesticide was completely protected from biological degradation and that sorbed- and solution-phase bacteria-degraded solution-phase pesticide, we found that the most strongly adsorbed fungicide also degraded the fastest. Therefore, the sorbed pesticide undergoes chemical degradation. Kah et al. (2007) observed faster degradation of pesticides in soils with strong adsorption due to pesticide hydrolysis on the soil surface. *Step 4*: The daily input of water in the system can desorb the non-degraded pesticides toward the soil solution (according to the desorption percentage). *Step 5*: The desorbed pesticide moved toward the next soil layer according to the travel time of bromide, i.e., the transit of the fungicides to the next soil layer occurred after they had been adsorbed and degraded in the current layer. As such, the empirical model did not assume that non-reactive and reactive compounds have the same retardation factor (RF), but rather, EMOLP uses the travel time of bromide to move the available pesticide in the soil solution after the relevant chemical reactions have occurred. The time required for bromide (a non-reactive substance with RF = 1) to be detected at a specific soil depth was thus used to represent the travel time of the pesticide that did not react (Paraiba and Spadotto 2002)—in this case, the pesticide that desorbed after adsorption and degradation had already occurred. Step 6: The pesticide that remained in the soil solution was assumed to degrade at the same rate as the fungicide adsorbed onto soil solids (Fig. S1 in Supporting Information). Laboratory and field assays were carried out over one year of pesticide application to calibrate the parameters of the EMOLP (penetration depth during application of compounds directly on the soil, rate of degradation, and fraction of pesticide deposited in topsoil after foliar application). The mean square error (MSE) was used as an objective measure of model error.

#### Subsurface soil layers

The model for subsurface soil layers (20–100 cm) was the same as for the topsoil (steps 1–6), except that: (i) The input of the substance in a subsequent soil layer was related to the desorbed pesticide in the upper soil layer and the travel time of the conservative tracer between layers, and (ii) the degradation time in the first-order kinetic model was assumed to equal one day due to continuous desorption of the pesticide in the upper soil layer (continuum substance input in the subsequent soil layer). Therefore, the initial concentration in the first-order kinetic equation changed every day (Fig. S1 in Supporting Information). During the calibration of the empirical model at a depth of 20–40 cm, the degradation rate of the pesticide was evaluated using the MSE values. The EMOLP model could not be calibrated at a depth of 40–60 cm, 60–80 cm, and 80–100 cm because the experimental concentrations of the pesticides were below the detection limit of the method.

For all soil layers, the degradation rates and the adsorption and desorption percentages were determined experimentally under laboratory conditions, and the travel time of the conservative tracer was measured under field and laboratory conditions. The soil layers were assumed to be homogeneous and show a steady-state flow, and desorption was assumed to be constant over time.

#### Initial pesticide concentrations in surface and subsurface layers

The initial pesticide concentration in topsoil was calculated as:

4 $$ C_{0} = {\raise0.7ex\hbox{${D_{{{\text{app}}}} f_{{{\text{soil}}}} * 10^{ - 4} {\text{ha}}/{\text{m}}^{2} }$} \!\mathord{\left/ {\vphantom {{D_{{{\text{app}}}} f_{{{\text{soil}}}} * 10^{ - 4} {\text{ha}}/{\text{m}}^{2} } {P_{{{\text{dp}}}} * \rho_{b} * 1000\;{\text{L}}/{\text{m}}^{3} }}}\right.\kern-0pt} \!\lower0.7ex\hbox{${P_{{{\text{dp}}}} * \rho_{b} * 1000\;{\text{L}}/{\text{m}}^{3} }$}} $$

where $C_{0}$ is the initial concentration (mg a.i. kg^−1^ dry soil), $D_{{{\text{app}}}}$ is the applied dose of pesticides (mg ha^−1^), $f_{{{\text{soil}}}}$ is the fraction of pesticide deposited in topsoil during spraying, $P_{{{\text{dp}}}}$ is the penetration depth of pesticide in the soil (m), and $\rho_{b}$ is the soil bulk density (kg L^−1^) (Birkved and Hauschild 2006; Juraske et al. 2011). The initial concentration in the solid and liquid phases of the surface layer was calculated using the following proposed equations (Eqs. (5) and (6)):

5 $$ C_{0s\left( i \right)} = C_{0} * f_{{\% {\text{Ads}}_{z} }} $$

6 $$ C_{0w\left( i \right)} = C_{0} * \left( {1 - f_{{\% {\text{Ads}}_{z} }} } \right) * \rho_{bz} $$

where $C_{0s\left( i \right)}$ (mg kg^−1^) is the initial concentration of pesticide in the solid phase of the topsoil, $C_{0w\left( i \right)}$ (mg L^−1^) is the initial concentration of pesticide in the aqueous phase of the topsoil, $f_{{\% Ads_{z} }}$ is the fraction of adsorption percentage in soil layer ${\text{z}}$, and $\rho_{bz}$ is the bulk density in soil layer ${\text{z}}$. Equations (7) and (8) were used to estimate the initial concentrations in soil solid and liquid phases for subsurface layers:

7 $$ C_{0ss} = C_{{0{\text{sub}}}} *f_{{\% {\text{Ads}}_{z} }} * \left( {1/\rho_{bz} } \right) $$

8 $$ C_{0ws} = C_{{0{\text{sub}}}} *\left( {1 - f_{{\% {\text{Ads}}_{z} }} } \right) $$

where $C_{0ss}$ (mg kg^−1^) is the initial concentration of pesticide in the solid phase of the subsurface soil layers, $C_{0ws}$ (mg L^−1^) is the initial concentration of pesticide in the aqueous phase of the subsurface soil layers, and $C_{0sub}$ is the pesticide concentration (mg L^−1^) in the aqueous soil solution related to the desorbed pesticide from the upper soil layer. Equation (4) was not used to calculate the initial concentration in the subsurface soil layers because the pesticide applications (soil and foliar) were only carried out in the topsoil (0–20 cm).

#### Time-dependent soil solid and liquid pesticide concentrations in surface and subsurface layers

The time-dependent concentrations of the pesticide in soil solids and aqueous solution were described using the following proposed equations (Eqs. (9)–(12)):

9 $$ \begin{array}{*{20}c} {C_{t\left( 1 \right)s} = \left[ {\left( {C*e^{{ - k_{z} t}} } \right) - \left( {\left( {C*e^{{ - k_{z} t}} } \right) * f_{{\% Des_{z} }} } \right) + C} \right]} \\ {C_{t\left( 2 \right)s} = \left[ {\left( { C_{t\left( 1 \right)s} *e^{{ - k_{z} t}} } \right) - \left( {\left( { C_{t\left( 1 \right)s} *e^{{ - k_{z} t}} } \right) * f_{{\% Des_{z} }} } \right) + C} \right]} \\ {C_{t\left( n \right)s} = \left[ {\left( {C_{{t\left( {n - 1} \right)s}} *e^{{ - k_{z} t}} } \right) - \left( {\left( {C_{t\left( n \right)s} *e^{{ - k_{z} t}} } \right)* f_{{\% Des_{z} }} } \right) + C} \right]} \\ \end{array} $$

10 $$ \begin{array}{*{20}c} {C_{t\left( 1 \right)w} = \left[ {\left( {C_{0w\left( i \right)} * e^{{ - k_{z} t}} } \right)} \right] } \\ {C_{t\left( 2 \right)w} = \left[ {\left( {C_{0w\left( i \right)} *e^{{ - k_{z} t}} } \right)} \right] } \\ {C_{t\left( n \right)w} = \left[ {\left( {C_{0w\left( i \right)} *e^{{ - k_{zt} }} } \right)} \right]} \\ \end{array} or \begin{array}{*{20}c} {C_{t\left( 1 \right)w} = \left[ {\left( {C_{0w\left( f \right)} *e^{{ - k_{z} t}} } \right)} \right]} \\ {C_{t\left( 2 \right)w} = \left[ {\left( {C_{0w\left( f \right)} *e^{{ - k_{z} t}} } \right)} \right]} \\ {C_{t\left( n \right)w} = \left[ {\left( {C_{0w\left( f \right)} *e^{{ - k_{z} t}} } \right)} \right]} \\ \end{array} $$

11 $$\begin{array}{c}{C}_{t\left(1\right)w}= \left[\left({C}_{0ws}* {e}^{-{k}_{z}*1}\right)+ \left(\left(\left({C}_{0ss}*{e}^{-{k}_{z}*1}\right) * {f}_{{\%Des}_{z}}\right) * {\rho }_{bz}\right)+ {C}_{0ws}\right] \\ {C}_{t\left(2\right)w}= \left[\left({C}_{t(1)w}* {e}^{-{k}_{z}*1}\right)+\left(\left(\left({ C}_{t\left(1\right)s}* {e}^{-{k}_{z}*1}\right) * {f}_{{\%Des}_{z}}\right)* {\rho }_{bz}\right)+ {C}_{0ws}\right] \\ {C}_{t\left(n\right)w}= \left[\left({C}_{t(n-1)w}*{e}^{-{k}_{z}*1}\right)+ \left(\left(\left({C}_{t\left(n-1\right)s} {e}^{-{k}_{z}*1}\right)* {f}_{{\%Des}_{z}}\right)* {\rho }_{bz}\right)+ {C}_{0ws}\right]\end{array}$$

12 $$ \begin{array}{*{20}c} {C_{t\left( 1 \right)w} = \left[ {\left( {C_{0ws} * e^{{ - k_{z} *1}} } \right) + C_{0ws} } \right]} \\ {C_{t\left( 2 \right)w} = \left[ {\left( {C_{t\left( 1 \right)w} * e^{{ - k_{z} *1}} } \right) + C_{0ws} } \right]} \\ {C_{t\left( n \right)w} = \left[ {\left( {C_{{t\left( {n - 1} \right)w}} * e^{{ - k_{z} *1}} } \right) + C_{0ws} } \right]} \\ \end{array} $$

where $C_{t\left( n \right),s}$ is the pesticide concentration in soil solid (mg kg^−1^) at time *t*, $f_{{\% Des_{z} }}$ is the fraction of desorption percentage in soil layer ${\text{z}}$, $C_{t\left( n \right),w}$ is the pesticide concentration in the soil liquid phase (mg L^−1^) at time *t*,${ }k_{z}$ is the degradation rate of pesticide in the soil layer ${\text{z}}$, ${\text{C}}$ is $C_{0s\left( i \right)}$ (topsoil) or $C_{0ss}$ (subsurface soil layers). In the topsoil, $C_{{t\left( {n - 1} \right)s}}$ is equal to $C_{0s\left( i \right)}$, and $C_{0s\left( i \right)}$ changes according to pesticide applications. $C_{0w\left( f \right)}$ (mg L^−1^) is the concentration of pesticide in the aqueous phase of the topsoil after new pesticide applications. The variable $C_{0w\left( f \right)}$ takes into account the degradation of $C_{0w\left( i \right)}$ and the new inputs of pesticides in the soil ($C_{0w\left( n \right)}$). Equations (10) and (11) were used to estimate $C_{t\left( n \right)w}$ in the topsoil and the subsurface soil layers, respectively. The variable $\left( {\left( {\left( {C_{{t\left( {n - 1} \right)s}} e^{{ - k_{z} *1}} } \right)* f_{{\% Des_{z} }} } \right)* \rho_{bz} } \right)$ in Eq. (11) depends on the travel time of the conservative tracer. Once the pesticide desorbed from one layer and reached the next layer, this portion of the equation was no longer relevant, so Eq. (11) was simplified to Eq. (12). The laboratory assays to obtain local parameters and the field trials to calibrate, validate, and evaluate the EMOLP are summarized in the following sections.

### Field and column trials to calibrate, validate, and evaluate the EMOLP model

Field (dimethomorph and pyrimethanil quantification at different depths in the soil and groundwater samples) and column assays over one year of pesticide application were conducted to calibrate, validate, and evaluate the new empirical model EMOLP.

#### Field trials

The field trials were carried out inside the greenhouse in areas that measured 980 m^2^ (T-1) and 298 m^2^ (T-2). Dimethomorph and pyrimethanil were applied to the soil, leaves, and flower buds using motorized equipment (Bean® Pump, Model No. R-10). The percentage of the fungicides that reached the soil surface after foliar application, a drift assay, detailed dates and doses of fungicides, and bromide used over one year of cultivation are provided in Supporting Information.

Dimethomorph and bromide were applied once to the soil at T-1 to calibrate and at T-2 to validate the EMOLP model. Additionally, pyrimethanil was applied to the soil of T-2 to assess the robustness of the EMOLP with different parameters such as ${f}_{\mathrm{soil}}$, ${f}_{{\%\mathrm{Ads}}_{z}}$, and $k$. After a pulse application of Forum®, Scala®, and KBr (conservative tracer) to the soil, three soil profiles at depths of 0–20 cm, 20–40 cm, 40–60 cm, 60–80 cm, and 80–100 cm were collected on days 1, 3, 7, 17, 28, 42, 63, 91, 141, 244, and 364 in T-1. In T-2, the soil profiles were collected on days 1, 3, 9, 16, 30, 44, 58, 91, 142, 255, and 394. A randomized sampling scheme was applied after dividing the T-1 into 176 cells and T-2 into 48 cells, each with an area of 3.2 m^2^.

All of the soil samples were dried in foil containers at room temperature (18 °C) for three days, and 10.0 g of the samples was shaken with ethyl acetate (20 mL) to extract the pesticides, centrifuged at 7000 r min^−1^, and concentrated. The organic phase was injected into the CG-MS system (Mosquera-Vivas et al. 2016a, b). To obtain the conservative tracer, 10.0 g of dry soil was extracted with 20.0 mL of Milli-Q water and shaken. The aqueous extracts were centrifuged at 8500 r min^−1^ for 15 min, filtered (0.22 μm), and injected into the HPLC–DAD system. The limit of detection (LOD) and limit of quantification (LOQ) of bromide were estimated to be 0.55 mg kg^−1^ and 1.83 mg kg^−1^, respectively. The recovery of bromide was 89.4% with a coefficient of variation of 11.7%. Three additional soil profiles for T-1 and T-2 were analyzed before pulse application to determine whether dimethomorph, pyrimethanil, and bromide residues were present in the soil.

The groundwater table in T-1 and T-2 was between 1.00 and 1.50 m. Nine groundwater samples were extracted for T-1 and seven for T-2 during the field assays. A 500 mL groundwater sample was filtered through 0.45-μm filters. Then, dimethomorph and pyrimethanil were obtained using solid-phase extraction (SFE) with LiChrolut EN (200 mg) cartridges. Methanol and ethyl acetate 1:1 v/v were added to elute the compounds. The organic extracts were evaporated at 35 °C, diluted to 1 mL with ethyl acetate, and injected into the GC–MS system. In addition, an aliquot of filtered groundwater was injected into the HPLC–DAD system to determine the concentration of the conservative tracer.

#### Column assays

We collected an undisturbed soil column between 0 and 1 m (defined based on the depth of the groundwater table) to confirm the travel time of bromide (conservative tracer) in the field trials. The “Water flow travel time based on the travel time of the conservative tracer” section describes the method we used to evaluate the travel time of bromide in the soil column. In addition, two undisturbed soil columns between 0 and 20 cm were taken to obtain the penetration depth of pesticides after application directly to the soil.

##### Water flow travel time based on the travel time of the conservative tracer

Bromide is one of the most widely used conservative tracers worldwide to study water and solute transport under field and laboratory conditions (Haddad et al. 2019). Estimating the travel time (the time required for a compound to cross the soil profile) of a non-reaction substance using the concept of convection time (Juraske et al. 2011; Rao et al. 1985) leads to the underestimation of pesticide transport through the soil profile (Eq. (S8) in Supporting Information); therefore, the arrival time of the conservative tracer was obtained experimentally using an undisturbed soil column between 0 and 1 m (Fig. S3 in Supporting Information). The time required for bromide (a non-reactive substance with RF = 1) to be detected at a specific soil depth was considered the non-reaction pesticide travel time. The undisturbed soil column conditions and properties are summarized in Supporting Information.

To obtain the travel time of the conservative tracer to a depth of 20 cm, 40 cm, 60 cm, and 80 cm, 0.01 M CaCl_2_ solution was applied with trickle irrigation to the top of the column until steady-state flow was achieved. A step input with 581 mg L^−1^ of bromide was applied daily with trickle irrigation for 216 days, and the soil solution was collected in each effluent sampling point over time. The aqueous samples were filtered (0.22 μm) and injected into the HPLC–DAD system. Details of the bromide quantification are provided in Supporting Information.

##### Penetration depth during application of fungicides directly onto the soil

The two undisturbed soil columns, 15 cm in diameter and 20 cm long, were held at an average temperature of 20.0 °C ± 1.0 °C and average relative humidity of 44.0% ± 4.0%. The daily soil temperature was 18 °C ± 1.0 °C at 5 cm depth. An effluent sampling point was located throughout the length of the soil column at a height of 12 cm and a final outlet sampling point. We applied 57 mL of 0.01 M CaCl_2_ solution with trickle irrigation to the top of the column until steady-state flow was achieved. A pulse of bromide (5321 mg L^−1^), dimethomorph (329 mg L^−1^), and pyrimethanil (515 mg L^−1^) was applied on the surface of the soil, and then 57 mL of 0.01 M CaCl_2_ solution (3,2 mm at 8 mL min^−1^) was applied with trickle irrigation for 190 days. The soil solution was collected on days 1, 3, 7, 14, 21, 30, 44, and 60 at the effluent sampling point at 12 cm depth and each day at the final outlet sampling point. All of the aqueous samples were filtered (0.22 μm) and injected into the HPLC–DAD system to quantify the bromide, and the soil solutions of the sampling point at 12 cm depth were extracted with ethyl acetate and injected into the GC–MS system to determine the concentrations of dimethomorph and pyrimethanil fungicides.

## Results and discussion

### Empirical model to asses leaching of pesticides—EMOLP

#### Chemical reaction of the fungicides

The retention parameters of the pesticides for the Andisol soil profile are summarized in Table 2. The linear and Freundlich models fit all of the adsorption data, as evidenced by coefficients of determination ${R}^{2}$ that ranged from 0.94 to 0.99. All of the fungicides showed isotherms classified as L-type in all soil layers ($na$ < 1), except for dimethomorph in soil layer SL1 ($na$ > 1, S-type isotherm). L-type isotherms indicate that there is a strong interaction between the soil solid phase (adsorbent) and adsorbate and that the pesticides are interacting with some lower-energy sites, as the high-energy sites may become saturated when the initial concentration is increased (Delle Site 2001). These types of isotherms have been previously reported for pyrimethanil and other pesticides in temperate soils (Capri et al. 2001; Liang et al. 2011a; Rojas et al. 2013; Yu et al. 2010; Valderde-García et al. 1992). Linear and S-type isotherms have also been described for the adsorption of pyrimethanil in soils (Capri et al. 2001). S-type isotherms demonstrate strong adsorption of the solvent onto the soil solid phase (Delle Site 2001).

**Table 2:** Adsorption parameters of fungicides for the Andisol soil profile

Pesticide	Soil type	Soil layer—depth (cm)	Linear model		${K}_{oc}$ ^*a*^	Freundlich model			${K}_{foc}$ ^*b*^	Percentage of adsorption, %ads^*c*^	${\Delta H}_{ads}$, kJ mole^−1^	${\Delta G}_{ads}^{^\circ }$, kJ mole^−1^
			${K}_{d}$, L kg^−1^	R^2^	$\left(\frac{{K}_{d}}{\mathrm{\%CO}}\right)*100$, L kg^−1^	${K}_{fa}$	$na$	R^2^	$\left(\frac{{K}_{fa}}{\mathrm{\%CO}}\right)*100$			
Pyrimethanil*	Andisol	SL1 (0–20)	119.1 ± 6.4	0.98	1153	85.8 ± 10.5	0.78 ± 0.06	0.98	830	98.8 ± 0.5	− 23.35 ± 1.82	− 14.51 ± 1.06
		SL2 (20–40)	170.4 ± 9.2	0.98	1593	121.7 ± 20.4	0.81 ± 0.06	0.98	1137	99.1 ± 0.4	− 24.78 ± 2.21	− 15.46 ± 1.29
		SL3 (40–60)	171.8 ± 7.6	0.98	1784	130.6 ± 19.8	0.84 ± 0.06	0.98	1356	99.1 ± 0.4	− 25.08 ± 2.08	− 15.46 ± 1.21
		SL2-SL3 (20–60)^*d*^	171.1 ± 5.7^e^	0.98	1683^e^	126.0 ± 13.5^*e*^	0.83 ± 0.04^*e*^	0.98	1240^*e*^	99.1 ± 0.4^*e*^	–	–
		SL4 (60–80)	31.5 ± 1.5	0.98	651	29.8 ± 0.9	0.74 ± 0.03	0.99	615	95.5 ± 1.4	− 20.90 ± 1.28	− 11.54 ± 0.75
		SL5 (80–100)	8.1 ± 0.6	0.96	299	10.6 ± 0.4	0.72 ± 0.04	0.98	392	84.5 ± 3.5	− 17.44 ± 1.03	− 8.29 ± 0.60
Dimethomorph**	Andisol	SL1 (0–20)	35.3 ± 1.9	0.98	341	48.0 ± 6.2	1.26 ± 0.10	0.99	482	93.3 ± 1.7	− 15.77 ± 1.11	− 10.07 ± 0.65
		SL2 (20–40)	27.1 ± 0.8	0.99	253	28.0 ± 1.8	1.02 ± 0.06	0.99	263	93.0 ± 2.0	− 15.51 ± 1.44	− 10.05 ± 0.84
		SL3 (40–60)	17.1 ± 1.5	0.94	178	16.2 ± 1.7	0.86 ± 0.11	0.95	190	90.3 ± 2.3	− 14.37 ± 1.12	− 9.20 ± 0.66
		SL4 (60–80)	7.0 ± 0.5	0.96	145	7.2 ± 0.4	1.06 ± 0.16	0.96	148	78.6 ± 4.1	− 13.44 ± 0.94	− 7.17 ± 0.55
		SL5 (80–100)	4.5 ± 0.1	0.99	165	4.6 ± 0.1	0.94 ± 0.04	0.99	171	67.5 ± 1.7	− 13.35 ± 0.37	− 5.90 ± 0.22

^*a*^$K_{oc}$ is the coefficient of distribution normalized by fraction of OC content; ^b^$K_{foc}$ is the Freundlich coefficient of distribution normalized by fraction of OC content; ^*c*^ average of all individual points for each depth.${\text{\% Ads}} = {\text{mg adsorbed*}}100{\text{\% /mg spiked}}$; ^*d*^ adsorption parameters of this layer were calculated with data on soils from S2 (20–40 cm) and S3 (40–60 cm) together; ^*e*^$K_{fa}$, ${\text{na}}$, and $K_{d}$ values obtained were not statistically different between S2 and S3 (*p* > 0.05)

*The observed (adsorption) and estimated (linear adsorption) concentration data of pyrimethanil are shown in Figure S7 (Supporting Information; n = 10)

**The observed (adsorption) and estimated (linear adsorption) concentration data of dimethomorph are shown in Figure S8 (Supporting Information; n = 10)

The ± sing is the standard deviation of mean

In this study, the linear and Freundlich equations explained the experimental adsorption data of the fungicides because the adsorption energy values $na$ ranged from 0.75 to 0.95 and the ${C}_{\mathrm{eq}}$ values were below one-half of the substances’ water solubility (Delle Site 2001). Thus, the linear equation was used in the EMOLP instead of the Freundlich model. The linear model is the simplest and most widely used adsorption isotherm equation (Goldberg and Brown 2005). Furthermore, linear isotherms over a wide range of initial concentrations of pesticides suggest that all of the adsorption sites have equal energy for interacting with adsorbents and allow the use of adsorption percentages ($\%\mathrm{ads}$) rather than ${K}_{d}$ values in the EMOLP model. However, some authors reported that the Freundlich model allows the lowest error values in the isotherms optimizations of pesticides and represents the heterogeneity of the soil (Delle Site 2001; Khandelwal et al. 2020).

Most of the estimated Freundlich ${K}_{fa}$ and distribution ${K}_{d}$ (linear) coefficients of the fungicides in the Andisol were very similar to each other in the layers of the soil profile (Table 2). This is likely because both models fit the experimental adsorption data well, as mentioned above. The $\%\mathrm{ads}$, ${K}_{\mathrm{fa}}$, and ${K}_{\mathrm{d}}$ values of pyrimethanil and dimethomorph showed a marked decrease with depth (Table 2) as OC and clay content decreased. The adsorption coefficients were positively correlated with % OC (*r* between 0.95 and 0.88) and clay content (*r* between 0.91 and 0.89). Similar trends have been observed in European soils (Capri et al. 2001) and Australian soils (Yu et al. 2010) amended with biochars; in both cases, the adsorption of pyrimethanil was reported to increase with increasing OC and clay content. The behavior of the adsorption coefficients of dimethomorph can also be explained by the decrease in the effective cation exchange capacity ECEC (*r* = 0.99).

The adsorption of both fungicides in all soil layers was found to be an exothermic and spontaneous process, with ${\Delta H}_{ads}$ and ${\Delta G}_{ads}^{^\circ }$ on the order of 5.9–25.1 kJ mol^−1^ (Table 2). Pyrimethanil showed the highest $\mathrm{\%ads}$ owing to relatively weak physical forces, like aromatic (π–π interactions), electrostatic, and hydrogen bond interactions (Singh and Srivastava 2009). π-π interactions occur between the phenyl group of pyrimethanil and the aromatic components of the SOM (Mosquera-Vivas et al. 2016a). Electrostatic interaction occurs between the positive charges of pyrimidine and the net negative surface charge of soil. Hydrogen bond interaction occurs through the three acceptors and the one donor H-bond. Moreover, the fungicide pyrimethanil is a weak base (${pK}_{a}$ = 3.52 at 25 °C) and can be easily protonated under acidic conditions, such that it was mostly retained in soil layers SL2-SL3 (Table 2). The pH decreased from soil layers SL1 to SL2-SL3; thus, the protonated fungicide increased from 0.17% to 2.05% (Eq. (S6) in Supporting Information). The increase in protonated pyrimethanil in soil layers SL2 and SL3 allowed an electrostatic interaction with the net negative surface charge of the soil and could be easily adsorbed, leading to increased ${K}_{\mathrm{fa}}$ and ${K}_{\mathrm{d}}$ coefficients. Dimethomorph was 100% deprotonated (Eq. (S5) in Supporting Information) in all soil layers, and the oxygen atoms in its chemical structure attract electron density (electronegativity); therefore, the polar carbon type (carbonyl-C) of the SOM (Mosquera-Vivas et al. 2016a) repels the fungicide and thus decreased fungicide adsorption in the soil. The negative charges formed by the dipole (oxygen atoms) may explain the correlation found between the adsorption coefficients and ECEC, since the divalent cations Ca^2+^ and Mg^2+^ allow soil-divalent cation-dimethomorph linkages.

Pyrimethanil ${K}_{foc}$ values ranged from 1240 to 392 and ${K}_{oc}$ values from 1683 L kg^−1^ to 299 L kg^−1^. The range of ${K}_{foc}$ was within the range reported in the literature for temperate soils (Capri et al. 2001; PPDB 2022; Vanni et al. 2006). However, the range of ${K}_{oc}$ here was higher than that published by Langeron et al. (2014). In general, ${K}_{foc}$ (482–148) and ${K}_{oc}$ (341 L kg^−1^–145 L kg^−1^) values of dimethomorph showed higher adsorption in our soil than in temperate soils (Maillard et al. 2011).

The linear and Freundlich single-point desorption parameters and apparent desorption hysteresis ($HI$) for the pesticides in the Andisol soil profile are shown in Table 3. The distribution coefficients of desorption (${K}_{d24}$ and ${K}_{fd24}$) were higher and significantly different (*p* < 0.05) from the distribution coefficient of adsorption (${K}_{\mathrm{d}}$ and ${K}_{\mathrm{fa}}$). The apparent desorption hysteresis of pyrimethanil ($\mathrm{HI}$ values between 0.88 and 2.30) and dimethomorph (($\mathrm{HI}$ values between 0.80 and 1.40) indicated that pesticide mass transfer from soil solution to solid (adsorption) occurs more readily than solute mass transfer from soil solid to the solution with single-step desorption (Mosquera-Vivas et al. 2016a) due to the adsorption intensity and the amount of soil organic matter. Mamy and Barriuso (2007) reported that ${K}_{\mathrm{fa}}$ coefficients > 20 allowed pesticide transfers from reversible to irreversible adsorption sites. Therefore, the adsorption process of the substance showed hysteresis. In addition, the increase of pesticide desorption percentages with increasing soil depth (Table 3) was negatively correlated with the organic carbon content (*r* between − 0.99 and − 0.90), suggesting that the amount of pesticides desorbed decreased as the OC content in the soil increased (Yu et al. 2006; Yu et al. 2010). Therefore, the vertical transport of the fungicide increased with depth in the soil profile, and dimethomorph had the highest risk of polluting groundwater. The adsorption–desorption percentages, as a part of the chemical reaction of the EMOLP, allowed us to estimate the pesticide concentrations in soil solid and soil solution at different depths over time, according to the calibration, validation, and evaluation of the empirical model described below.

**Table 3:** Single-point desorption parameters of fungicides for the Andisol soil profile

Pesticide	Soil type	Soil layer depth (cm)	Linear model		Freundlich model			Percentage of Desorption, $\mathrm{\% des}$ ^*a*^	HI index
			${K}_{d24}$*,* L kg^−1^	R^2^	${K}_{fd24}$	$nd24$	R^2^		
Pyrimethanil*	Andisol	SL1 (0–20)	251.5 ± 44.6	0.80	133.2 ± 84.6	0.73 ± 0.21	0.79	0.8 ± 0.6	1.11
		SL2 (20–40)	562.0 ± 30.0	0.98	323.1 ± 70.8	0.81 ± 0.06	0.98	0.4 ± 0.2	2.30
		SL3 (40–60)	419.5 ± 38.9	0.94	187.4 ± 50.2	0.70 ± 0.08	0.95	0.4 ± 0.2	1.44
		SL4 (60–80)	59.2 ± 4.6	0.95	43.9 ± 3.0	0.66 ± 0.04	0.98	2.5 ± 0.9	0.88
		SL5 (80–100)	19.6 ± 1.5	0.96	19.5 ± 0.9	0.75 ± 0.05	0.98	7.8 ± 1.1	1.42
Dimethomorph**	Andisol	SL1 (0–20)	66.3 ± 6.1	0.94	51.5 ± 11.8	0.85 ± 0.10	0.95	3.9 ± 0.7	0.88
		SL2 (20–40)	65.3 ± .2	0.97	50.1 ± 7.6	0.84 ± 0.07	0.98	3.7 ± 0.6	1.40
		SL3 (40–60)	35.2 ± .6	0.96	28.3 ± 3.6	0.81 ± 0.03	0.97	5.8 ± 0.8	1.06
		SL4 (60–80)	12.6 ± .4	0.99	12.1 ± 0.4	0.89 ± 0.03	1.00	13.1 ± 1.0	0.80
		SL5 (80–100)	9.4 ± 0.9	0.93	8.9 ± 0.9	0.91 ± 0.12	0.94	17.7 ± 4.0	1.08

^*a*^ % desorption was calculated as: $\mathrm{\% des}=\mathrm{\%ads}-{\left(\mathrm{\%ads}\right)}_{24}$ and $HI= \frac{{K}_{d24}}{{K}_{d}}-1$ (Huang et al. 1998)

*The observed (single-point desorption) and estimated (linear desorption) concentration data of pyrimethanil are shown in Figure S7 (Supporting Information; n = 10)

**The observed (single-point desorption) and estimated (linear desorption) concentration data of dimethomorph are shown in Figure S8 (Supporting Information; n = 10)

The ± sing is the standard deviation of mean

The current manuscript reports, for the first time, the degradation process of the fungicides dimethomorph and pyrimethanil through a tropical soil profile. The degradation rate ($k$) and the half-life (${t}_{(1/2)}$) obtained by fitting a first-order kinetic model to dimethomorph and pyrimethanil data from the Andisol are given in Table 4. The model performed well, with R^2^ values between 0.69 and 0.99. The null hypothesis (H_0_: $k=0$ and ${C}_{0}=0$) was rejected at a 95% confidence level, except for dimethomorph in soil layer SL5 (*R*^2^ = 0.17, *p* = 0.306). The poorer fit obtained for dimethomorph in soil layer SL5 was because it degraded to nearly 13.78% of its maximum concentration after 102 days of incubation. The $k$ values of dimethomorph (0.039 d^−1^–0.009 d^−1^) and pyrimethanil (0.0053 d^−1^–0.004 d^−1^) decreased from soil layers SL1 to SL2, then remained constant in soil layers SL2–SL4, and reached its minimum in soil layer SL5 (Table 4). This could be explained by the decrease in microbial activity (respiration) with depth in the soil profile. For instance, the amount of CO_2_ evolved during incubation studies was found to decrease in soil layers SL1-SL3, after which it was constant; accordingly, the decrease in microbial activity allowed the $k$ values of the fungicides to decrease (Mosquera-Vivas et al. 2016b). Bio-degradation plays an important role in the decrease of dimethomorph in soils, evidenced by the fact that the fungicide is stable under sterile conditions (FAO 2014a). However, pyrimethanil showed the highest ${K}_{d}$ and $k$ values in soil layer SL1, indicating that the more strongly adsorbed fungicide also degraded the fastest. Kah et al. (2007) observed a faster degradation of metsulfuron-methyl and pirimicarb in soils with strong adsorption because of their hydrolysis on the soil surface. Hence, biotic and abiotic processes might explain pyrimethanil degradation within the first 20 cm of the Andisol. Measurements of secondary metabolites of the fungicides are needed to address the fundamental mechanisms of biotic and abiotic degradation.

**Table 4:** Degradation parameters of fungicides for the Andisol soil profile

Pesticide	Soil type	Soil layer depth (cm)	First-order kinetic			
			$-k$ ^*a*^*,* d^−1^	${t}_{(1/2)}$ ^*b*^*,* d	R^2* c*^	p^*d*^
Pyrimethanil*	Andisol	SL1 (0–20)	0.053 ± 0.009	13.1	0.86	0.001
		SL2 (20–40)	0.013 ± 0.002	49.5	0.84	0.0001
		SL3 (40–60)	0.010 ± 0.001	69.3	0.91	0.001
		SL4 (60–80)	0.008 ± 0.002	86.6	0.79	0.003
		SL5 (80–100)	0.004 ± 0.001	173.3	0.69	0.006
Dimethomorph**	Andisol	SL1 (0–20)	0.039 ± 0.002	17.8	0.99	0.0001
		SL2 (20–40)	0.010 ± 0.001	69.3	0.90	0.0001
		SL3 (40–60)	0.009 ± 0.003	77.0	0.71	0.018
		SL4 (60–80)	0.009 ± 0.003	77.0	0.73	0.015
		SL5 (80–100)	0.001 ± 0.001	693.2	0.17	0.306

^*a*^ $$k$$ is the constant rate of the first-order kinetic; ^*b*^ $$t_{{\left( 
{1/2} \right)}}$$ is the half-life calculated such as: $$t_{1/2} = \ln 0.5/ - k$$; ^*c*^ coefficient of determination and ^*d*^ probability at a 95% confidence level

*The observed and estimated (first-order kinetic) concentration data of pyrimethanil are shown in Figure S9 (Supporting Information; n between 20 and 18)

**The observed and estimated (first-order kinetic) concentration data of dimethomorph are shown in Figure S10 (Supporting Information; n between 20 and 14)

The ± sing is the standard deviation of mean

The ${t}_{(1/2)}$ values of dimethomorph (17.8 d–77.0 d) and pyrimethanil (13.1 d–173.3 d) increased with soil depth, and these values for both fungicides in soil layer SL1 were shorter than those previously reported in temperate soils (Capri et al. 2001; FAO 2014a, b; Fenoll et al. 2011; Piao et al. 2011; PPDB 2022; Vanni et al. 2006), showing that dimethomorph and pyrimethanil degrade faster in tropical than in temperate soils. However, if the fungicides reach the deepest soil layer (80–100 cm), they might constitute a risk for groundwater pollution owing to their persistence and mobility, as shown above.

#### Calibration, validation, and evaluation of the EMOLP model

**Fig. 1:**
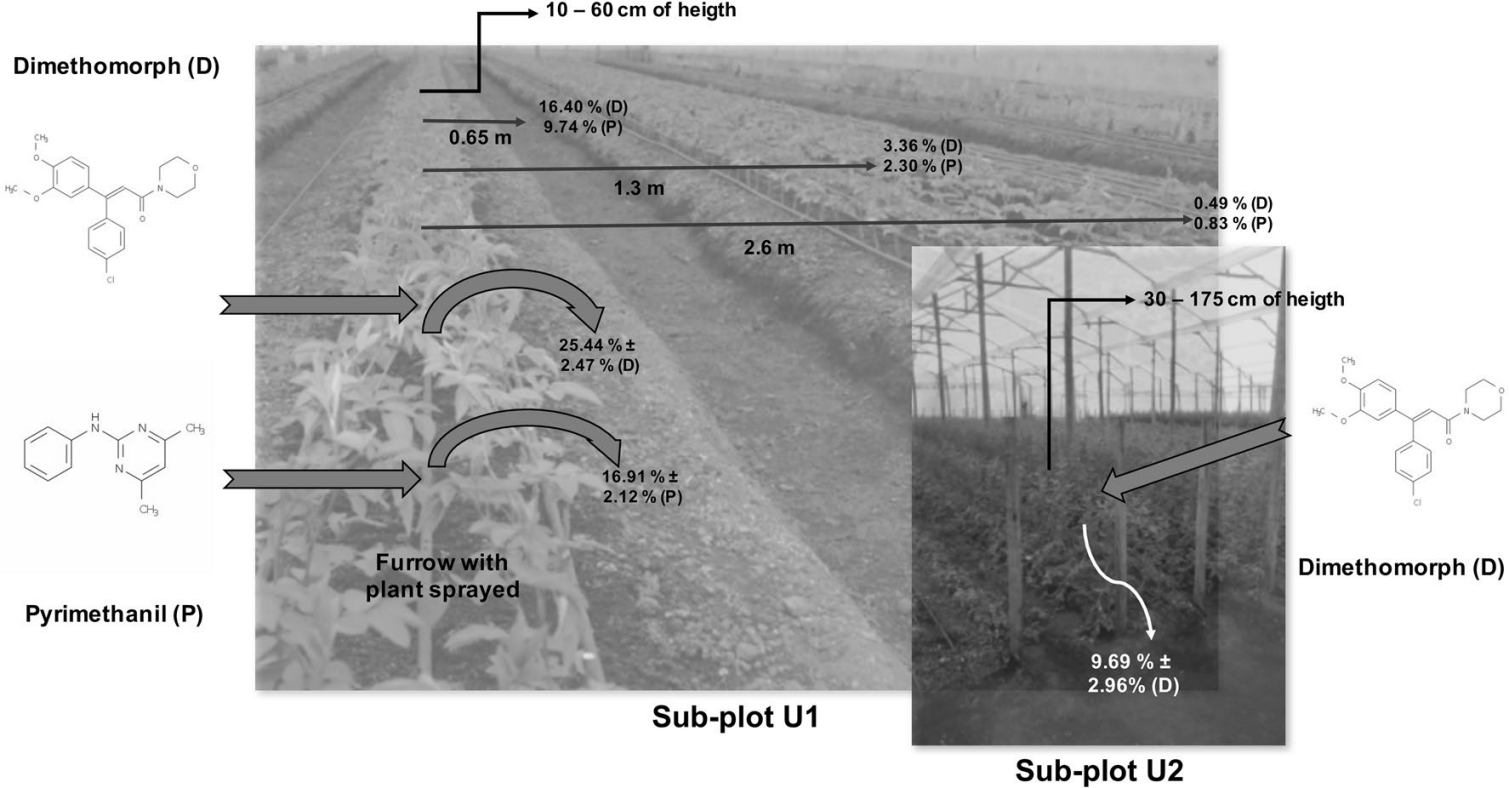
Fraction of fungicide deposited in topsoil after foliar application. The ± sing is the standard deviation of mean

Dimethomorph and pyrimethanil deposition and drift during foliar applications (Supporting Information) are shown in Fig. 1. The percentage of dimethomorph deposited on Andisol (${\%P}_{D}$) was 25.44% ± 2.47% in the sub-plot U1 and 9.69% ± 2.96% in the sub-plot U2. This indicates that the amount of fungicide that reached the soil depended upon the stage of plant growth; the amount of dimethomorph deposited was highest during earlier stages, when the plants are shorter (Fig. 1). The ${\%P}_{D}$ of pyrimethanil that reached the soil surface in sub-plot U1 (16.91% ± 2.12) was lower than the amount of dimethomorph. In the drift assay, 14.37% of the pyrimethanil sprayed on the plant reached the furrow where the plant was rooted; 9.74% reached a distance of 0.65 m, 2.30% reached 1.3 m, and 0.83% reached 2.6 m away. This is compared to 24.13%, 16,40%, 3,36%, and 0.49% of the dimethomorph at the same distances (Fig. 1). These differences could be because pyrimethanil has higher volatility than dimethomorph, allowing a higher amount of drift 2.6 m away. According to these results, ${f}_{soil}$ (Eq. (1)) used in the calibration, validation, and evaluation of the EMOLP model for the Andisol soil type was 0.2544 for dimethomorph and 0.1691 for pyrimethanil. These values represent the worst-case scenario, i.e., during the first stage of plant growth when deposition of the pesticides from foliar applications is highest.

**Fig. 2:**
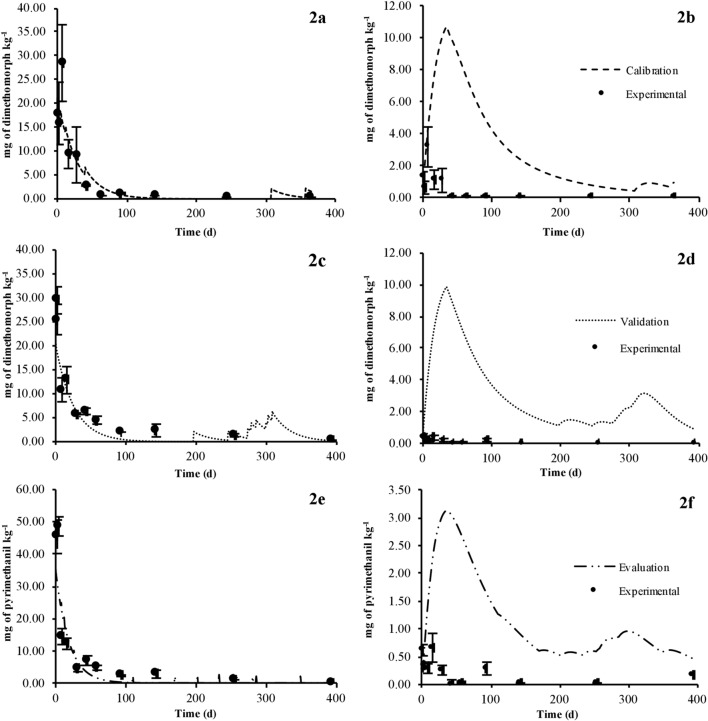
Calibration, validation, and evaluation of the Empirical Model of Leaching of Pesticides (EMOLP): calibration of the model with experimental data from the fungicide dimethomorph in T-1 at a depth of 0–20 (**a**) and 20–40 cm in T-1 (**b**). Validation of the model with experimental data from the fungicide dimethomorph in T-2 at a depth of 0–20 cm (**c**) and 20–40 cm (**d**). Evaluation of the model with experimental data from the fungicide pyrimethanil in T-2 at a depth of 0–20 cm (**e**) and 20–40 cm (**f**). The concentrations of the fungicides correspond to the total concentrations of the pesticides in the soil (the sum of the solid and aqueous concentrations). The bar is the standard deviation of mean

**Fig. 3:**
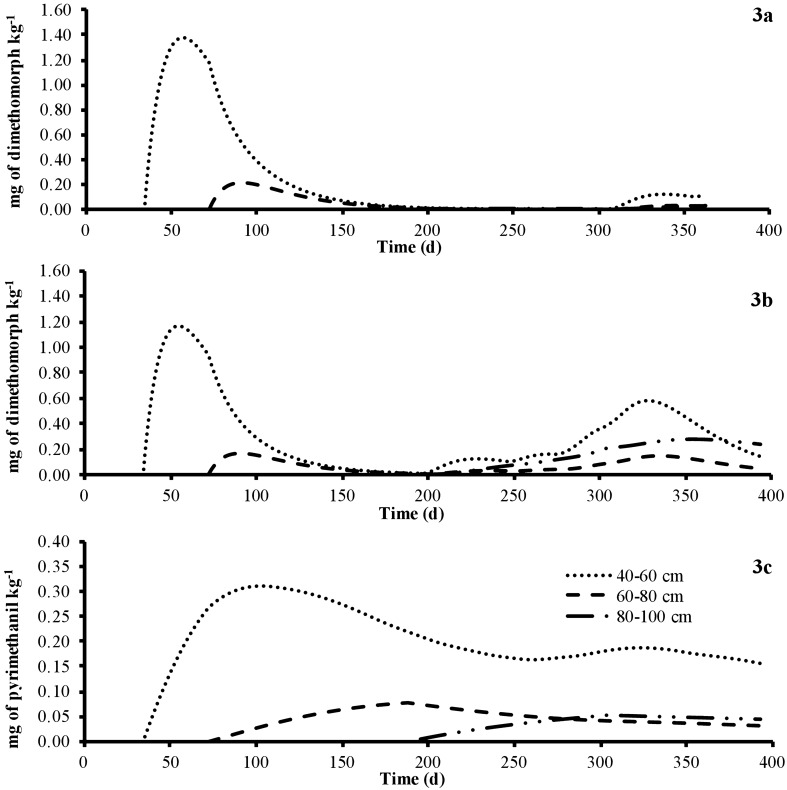
Simulation using the Empirical Model of Leaching of Pesticides (EMOLP) of dimethomorph and pyrimethanil concentrations in the subsurface layers of the soil profile: dimethomorph concentrations at depths of 40–60 cm, 60–80 cm, and 80–100 cm in T-1 (**a**), dimethomorph concentrations at depths of 40–60 cm, 60–80 cm, and 80–100 cm in T-2 (**b**), pyrimethanil concentrations at depths of 40–60 cm, 60–80 cm, and 80–100 cm in T-2 (**c**). The concentrations of the fungicides correspond to the total concentrations of the pesticides in the soil (the sum of the solid and aqueous concentrations)

All of the experimental parameters used to calculate the initial concentration and the time-dependent soil solid and liquid concentrations of the fungicide dimethomorph are summarized in Table 5, and the calibration and validation of EMOLP at depths of 0–20 cm and 20–40 cm are shown in Fig. 2. The simulated dimethomorph concentration at depths of 40–60 cm, 60–80 cm, and 80–100 cm is shown in Fig. 3. The concentrations of the fungicides presented in Figs. 2 and 3 correspond to the total concentrations of the pesticides in the soil (the sum of the solid and aqueous concentrations).

**Table 5:** Parameters for estimating the leaching of fungicides for the Andisol soil profile

Soil Type	Soil layer depth (cm)	$\rho_{b}$ ^a^, kg L^−1^	$f\% {\text{Ads}}$ ^b^		$f\% Des$ ^c^		$fsoil$ ^f^		$P_{{{\text{dp}}}}$ ^g^, m		$P_{dp}$ ^h^, m		$- k$ ^i^, d^−1^	
			Soil application						Foliar application					
			D ^*d*^	P ^*e*^	D ^*d*^	P ^*e*^	D ^*d*^	P ^*e*^	D ^*d*^	P ^*e*^	D ^*d*^	P ^*e*^	D ^*d*^	P ^*e*^
Andisol	SL1 (0–20)	0.48 ± 0.08	0.933	0.988	0.039	0.008	0.2544	0.1691	0.12	0.12	0.02	0.02	0.039	0.053
	SL2 (20–40)	0.57 ± 0.03	0.930	0.991	0.037	0.004	N.A	N.A	N.A	N.A	N.A	N.A	0.010	0.013
	SL3 (40–60)	0.64 ± 0.05	0.903	0.991	0.058	0.004	N.A	N.A	N.A	N.A	N.A	N.A	0.009	0.010
	SL4 (60–80)	0.71 ± 0.08	0.786	0.955	0.131	0.025	N.A	N.A	N.A	N.A	N.A	N.A	0.009	0.008
	SL5 (80–100)	0.67 ± 0.10	0.675	0.845	0.177	0.078	N.A	N.A	N.A	N.A	N.A	N.A	0.001	0.004

^a^ Bulk density values obtained with cylinder method; ^b^ fraction of adsorption percentage values obtained with batch method; ^c^ fraction of desorption percentage values obtained with batch method; ^d^ dimethomorph; ^e^ Pyrimethanil; ^f^ fraction of deposited fungicide in topsoil during spraying; ^g^ penetration depth of the pesticide during soil applications; ^h^ penetration depth of the pesticide during foliar application; ^I^ degradation rates in soil solid and aqueous solution, and N.A. is not apply

The ± sing is the standard deviation of mean

##### Calibration

During the calibration of the EMOLP using experimental data of the fungicide dimethomorph in T-1 at a depth of 0–20 cm, we found that the lowest mean square error value (MSE = 16 mg^2^ kg^−2^) was obtained with a penetration depth of the pesticide during soil applications equal to 0.12 m, the fraction of deposited pesticide in topsoil during spraying equal to 0.2544, penetration depth of the fungicide during foliar application equal to 0.02 m, and degradation rate in soil solid and aqueous solution equal to 0.039 d^−1^.

The penetration depth of dimethomorph and pyrimethanil during soil applications was assessed using undisturbed soil columns at a depth of 0–20 cm. The fungicides and bromide (conservative tracer) were detected in the soil solution one day after pulse application at a depth of 12 cm owing to low soil bulk density (Table 5), macroporosity, and advection flow of water in the soil. The concentration of bromide in soil solution decreased over time, while the dimethomorph and pyrimethanil concentrations increased until 7 days and then decreased over time (Fig. S5 in Supporting Information). Thus, this depth (12 cm) was considered in the calibration and validation of the EMOLP model. The advection flow of water in the soil and macroporosity was demonstrated by bromide breakthrough curves at a depth of 20 cm. According to Table S6 in Supporting Information, the convection–dispersion equation (CDE) in non-equilibrium better fit the experimental data with R^2^ and MSE values between 0.96 and 0.98 and 52 mg^2^ L^−2^–153 mg^2^ L^−2^, respectively. The adimensional partition coefficient between the mobile and immobile liquid phase ($\beta$) indicated that 43% (column 1) and 51% (column 2) of the water present in the soil resided in the matrix or slow zone of the soil. Furthermore, the transfer of mass between the two zones (mobile and immobile), $\omega$, allows the estimation of the average transfer time of water mass from the macropores to micropores (matrix) in the soil. If this time ($1/\omega$) is lower than the average travel time of bromide ($L/v$: $L$ is the length of the column and $v$ is the average pore-water velocity), the advection flow is predominant. The $L/v$ of the bromide in columns 1 and 2 was between 62.5 and 60.6 d, while the $1/\omega$ showed a range between 2.17 d and 1.75 d; therefore, the substances travel with the mass of water (advection or convection flow) through the macroporosity of the soil. The convection flow was further verified by the values of the Péclet number (${\mathrm{P}}_{\mathrm{e}})$ for columns 1 (5.19) and 2 (4.22). ${P}_{e}$ values greater than 1 mean that advection flow plays an important role in the transport of solutes compared to diffusive flow, though the presence of diffusive flow cannot be ruled out.

The penetration depth of the fungicides during foliar application is in accordance with Juraske et al. (2011), and the similarity of pesticide degradation rates in the solid and aqueous solution of the soil was demonstrated by the ${t}_{(1/2)}$ of dimethomorph in solid and solution at a depth between 0 and 20 cm. The degradation of dimethomorph in soil solution showed a first-order kinetic with a ${t}_{(1/2)}$ value equal to 12.6 d and a range of 9.4 d—19.3 d (Fig. S5 in Supporting Information). The ${t}_{(1/2)}$ value of the fungicide in solid soil was equal to 17.8 d (Table 4).

The calibration of the EMOLP at a depth of 20–40 cm showed an MSE value equal to 28 mg^2^ kg^−2^ using the degradation rate reported in Table 4. Interestingly, we obtained the lowest mean square error (MSE = 2 mg^2^ kg^−2^) with a degradation rate equal to 0.100 d^−1^ (ten times lower than reported in Table 4), indicating that pesticide degradation is a sensitive factor in the modeling of pollutants in the soil (Wolt et al. 2009). This optimized value was not used in the calibration process. Papadopoulou el at. (2016) reported that pesticides were more persistent under laboratory conditions than in the field. The uptake, volatilization, photolysis, and leaching of pesticides are likely considerably reduced in laboratory assays. However, these processes frequently occur during pesticide dissipation under field conditions (EEC 2000) both in the superficial and sub-superficial soil layers. The degradation rate under laboratory conditions at a depth of 20–40 cm thus probably overestimated the amount of dimethomorph over time by approximately threefold (Fig. 2). Therefore, the model demonstrates the need to carry out fungicide degradation and dissipation studies to establish a relationship between field and laboratory trials of these factors.

##### Validation

The calibrated empirical model was run to compare with the experimental data of the fungicide dimethomorph in T-2. MSE values of 56 mg^2^ kg^−2^ and 27 mg^2^ kg^−2^ were obtained at depths of 0–20 cm and 20–40 cm, respectively. Although the mean square error increased at a depth of 0–20 cm, the EMOLP model simulated the behavior of pesticides over time (Fig. 2) and demonstrated that it can be a useful mathematical tool to calculate pesticide concentrations in the soil (mainly at a depth of 0–20 cm) from values of chemical reaction under laboratory conditions. Again, the fungicide concentration at a depth of 20–40 cm was overestimated with the degradation rate under laboratory conditions, and the lowest MSE (3 mg^2^ kg^−2^) was achieved with a $k$ value ten times lower than reported in Table 5.

At depths of 40–60 cm, 60–80 cm, and 80–100 cm, most of the experimental concentrations of the fungicide dimethomorph were below the limit of detection (Table S7 and S8 in Supporting Information). The dimethomorph detected in the soil between 3 and 30 days in T-2 (Table S8 in Supporting Information) and in some groundwater samples at concentrations below 1 µg L^−1^ was due to pesticide application before the experiment began. This is confirmed by the behavior of the conservative tracer (bromide), which was not detected in T-1 and T-2 before the field trials but was detected 1 d after application at a depth of 20–40 cm, on day 43 at a depth of 40–60 cm, on day 93 at a depth of 60–80 cm, and day 255 at a depth 80–100 cm. It was never detected in the groundwater samples during the field trials. In the undisturbed soil column, bromide was detected 1d, 36 d, 74 d, and 190 d after application at 20 cm, 40 cm, 60 cm, and 80 cm (Fig. S4 in Supporting Information), respectively. It is possible that pesticides applied before the field trials reached the groundwater sources by preferential flow. During the field assays, preferential flow ways were evident in the study plot (Fig. S6 in Supporting Information). According to experimental concentrations of the fungicide dimethomorph at depths of 40–60 cm, 60–80 cm, and 80–100 cm in T-1 and T-2, the EMOLP model overestimated some amounts of the fungicide when using the degradation rates determined under laboratory conditions. The maximum overestimation was an order of magnitude at a depth of 40–60 cm (Fig. 3).

##### Evaluation

All of the experimental parameters used to calculate the initial concentration and the time-dependent soil solid and liquid concentrations of the fungicide pyrimethanil are summarized in Table 5. The experimental and simulated pyrimethanil concentrations at depths of 0–20 cm and 20–40 cm are shown in Fig. 2. The simulated pyrimethanil amounts at depths of 40–60 cm, 60–80 cm, and 80–100 cm are summarized in Fig. 3. During the evaluation of the EMOLP, MSE values of 61 mg^2^ kg^−2^ and 2.9 mg^2^ kg^−2^ were obtained at depths of 0–20 cm and 20–40 cm, respectively. The empirical model represented the behavior of pyrimethanil at both depths, but the $k$ value of fungicide under laboratory conditions at a depth of 20–40 cm led to an overestimation of the concentration (Fig. 2). The lowest MSE (0.1 mg^2^ kg^−2^) at a depth of 20–40 cm was achieved with a degradation rate ten times lower than reported in Table 5.

At depths of 40–60 cm, 60–80 cm, and 80–100 cm, most of the experimental concentrations of pyrimethanil were below the limit of detection (Table S8 in Supporting Information). The fungicide detected in the soil between 0 and 30 days in T-2 (Table S8 in Supporting Information) and in some groundwater samples at concentrations below 1 µg L^−1^ was due to pesticide application before the experiment began, as mentioned above. At these depths, the EMOLP model simulated very well the concentrations of pyrimethanil. For instance, at a depth of 40–60 cm, the experimental concentration of fungicide at 255 days was equal to 0.11 mg kg^−1^ ± 0.05 mg kg^−1^ and the simulated concentration with the EMOLP model was equal to 0.16 mg kg^−1^.

## Conclusion

The objective of our research was to develop a novel and reliable empirical model to simulate the leachability of pesticides in the first 100 cm of the soil profile. We used laboratory and field trials to calibrate, validate, and evaluate the empirical model EMOLP. The linear and Freundlich equations explained the adsorption data of dimethomorph (${K}_{oc}$: 145 L kg^−1^–341 L kg^−1^) and pyrimethanil (${K}_{\mathrm{oc}}$: 299 L kg^−1^–1683 L kg^−1^) in the Andisol soil profile. The degradation rates of the fungicides (first-order kinetic model) decreased from 0 to 40 cm and then remained constant in deeper soil layers (60–80 cm). At a depth of 80–100 cm, the fungicides dimethomorph and pyrimethanil showed less degradation; therefore, these pesticides could constitute a risk for groundwater pollution owing to their persistence and mobility. In this study, pyrimethanil showed the highest adsorption on the topsoil but had the shorter half-life (13,1 d), indicating that the more strongly adsorbed pesticide also degraded the fastest. The EMOLP is suited to simulating the pesticide concentration in soil solid and soil solution over time using adsorption–desorption percentage values and the degradation rates under laboratory conditions, mainly at a depth of 0–20 cm. The fungicide degradation rate at a depth of 20–40 cm was a critical input in the model, and the travel time of bromide (conservative tracer) allowed the fungicides to move throughout the soil profile. This provides strong evidence of the importance of estimating the chemical reactions of pesticides and the travel time of a conservative tracer at different soil layers to evaluate the leachability of compounds using the EMOLP model. The EMOLP could be further improved by including information on the relationship between dissipation (field) and degradation (laboratory) rates. The timescale of the EMOLP (1 day) performed adequately in terms of describing the chemical reaction. We suggest that EMOLP be used and validated in other tropical and temperate soils as an environmental assessment tool.

### Supplementary Information

Below is the link to the electronic supplementary material.

Supplementary file1 (DOCX 5396 KB)

Supplementary file1 (XLSX 285 KB)

## References

[CR1] Ahmad R, Kookana RS, Alston AM, Bromilow RH (2001). Differences in sorption behaviour of carbaryl and phosalone in soils from Australia, Pakistan, and the United Kingdom. Aust J Soil Res.

[CR2] Aslam S, Iqbal A, Lafolie F, Recous S, Benoit P, Garnier P (2018). Mulch of plant residues at the soil surface impact the leaching and persistence of pesticides: a modelling study from soil columns. J Contam Hydrol.

[CR4] Bannwarth MA, Sangchan W, Hugenschmidt C, Lamers M, Ingwersen J, Ziegler AD, Streck T (2014). Pesticide transport simulation in a tropical catchment by SWAT. Environ Pollut.

[CR5] Bernard H, Chabalier PF, Chopart JL, Legube B, Vauclin M (2005). Assessment of herbicide leaching risk in two tropical soils of reunion Island (France). J Environ Qual.

[CR6] Birkved M, Hauschild MZ (2006). PestLCI – A model for estimating field emissions of pesticides in agricultural LCA. Ecol Modell.

[CR7] Bragança I, Mucha AP, Tomasino MP, Santos F, Lemos PC, Delerue-Matos C, Domingues VF (2019). Deltamethrin impact in a cabbage planted soil: degradation and effect on microbial community structure. Chemosphere.

[CR8] Cabidoche Y-M, Achard R, Cattan P, Clermont-Dauphin C, Massat F, Sansoulet J (2009). Long-term pollution by chlordecone of tropical volcanic soils in the French West Indies: a simple leaching model accounts for current residue. Environ Pollut.

[CR9] Capri E, Camisa MG, Flores-Céspedes F, Glass CR, Gonzalez-Pradas E, Trevisan M (2001). Imidacloprid and pyrimethanil soil sorption. Agronomie.

[CR10] Cueff S, Alleto L, Bourdat-Deschamps M, Benoit P, Pot V (2020). Water and pesticide transfers in undisturbed soil columns sampled from a Stagnic Luvisol and a Vermic Umbrisol both cultivated under conventional and conservation agriculture. Geoderma.

[CR3] de Azeredo Morgado MG, Passos CJS, Garnier J, de Lima LA, de Alcântara MR, Samson-Brais E, Lucotte M (2023). Large-scale agriculture and environmental pollution of ground and surface water and sediment by pesticides in the Brazilian Amazon: the case of the santarém region. Water Air Soil Pollut.

[CR11] Delle Site A (2001). Factors affecting sorption of organic compounds in natural sorbent/water systems and sorption coefficients for selected pollutants. A review. J Phys Chem Ref Data.

[CR12] Demir AEA, Dilek FB, Yetis U (2019). A new screening index for pesticides leachability to groundwater. J Environ Manage.

[CR13] Dhuldhaj UP, Singh R, Singh VK (2023). Pesticide contamination in agro-ecosystems: toxicity, impacts, and bio-based management strategies. Environ Sci Pollut Res.

[CR14] Didoné EJ, Minella JPG, Tiecher T, Zanella R, Prestes OD, Evrard O (2021). Mobilization and transport of pesticides with runoff and suspended sediment during flooding events in an agricultural catchment of Southern Brazil. Environ Sci Pollut Res.

[CR15] Dotor-Robayo MY, Guerrero-Dallos JA, Martínez-Cordón MJ (2022). Influence of monoammonium phosphate on glyphosate adsorption-desorption in tropical soils: Effect of the order of sorbate additions. Chemosphere.

[CR16] European Economic Community (EEC) (2000) Guidance Document No 9188/VI/97-ver. 8, Persistence in Soil, Commission of the European Communities, Directorate General for Agriculture, GG VI B II-1, Brussels, Belgium

[CR17] Fang Y, Nie Z, Die Q, Tian Y, Liu F, He J, Huang Q (2017). Organochlorine pesticides in soil, air, and vegetation at and around a contaminated site in southwestern China: concentration, transmission, and risk evaluation. Chemosphere.

[CR18] FAO (Food and Agricultural Organization) (2014a) Dimethomorph (225). Available at https://www.fao.org/fileadmin/templates/agphome/documents/Pests_Pesticides/JMPR/Evaluation07/Dimethomorph.pdf. Accessed 06 September 2014a

[CR19] FAO (Food and Agricultural Organization) (2014b) Pyrimethanil (226) Available at https://www.fao.org/fileadmin/templates/agphome/documents/Pests_Pesticides/JMPR/Evaluation07/Pyrimethanil.pdf. Accessed 06 September 2014b

[CR20] Fenoll J, Ruiz E, Hellín P, Navarro S, Flores P (2010). Solarization and biosolarization enhance fungicide dissipation in the soil. Chemosphere.

[CR21] Fenoll J, Ruiz E, Flores P, Vela N, Hellín P, Navarro S (2011). Use of farming and agro-industrial wastes as versatile barriers in reducing pesticide leaching through soil columns. J Hazard Mater.

[CR22] Gámiz B, Velarde P, Spokas KA, Celis R, Cox L (2019). Changes in sorption and bioavailability of herbicides in soil amended with fresh and aged biochar. Geoderma.

[CR23] Gaona L, Bedmar F, Gianelli V, Faberi AJ, Angelini H (2019). Estimating the risk of groundwater contamination and environmental impact of pesticides in an agricultural basin in Argentina. Int J Environ Sci Technol.

[CR24] Goldberg S, Brown GE, Tabatabai MA, Sparks DL (2005). Equations and models describing adsorption processes in soils. Chemical processes in soils.

[CR25] Haddad K, Gheid A, Haddad D, Oulmi K (2019). Experimental and numerical study on the leaching of pesticides into the groundwater through a porous medium: effects of transport parameters. Environ Technol Innovation.

[CR26] Hall KE, Ray C, Ki SJ, Spokas KA, Koskinen WC (2015). Pesticide sorption and leaching potential on three Hawaiian soils. J Environ Manage.

[CR27] Heuvelink GBM, Burgers SLGE, Tiktak A, van den Berg F (2010). Uncertainty and stochastic sensitivity analysis of the GeoPEARL pesticide leaching model. Geoderma.

[CR29] Huang B, Yan D, Wang X, Wang X, Fang W, Zhang D, Ouyang C, Wang Q, Cao A (2019). Soil fumigation alters adsorption and degradation behavior of pesticides in soil. Environ Pollut.

[CR30] Huang W, Yu H, Weber WJ (1998). Hysteresis in the sorption and desorption of hydrophobic organic contaminants by soils and sediments: 1. A comparative analysis of experimental protocols. J Contam Hydrol.

[CR31] Jorda H, Huber K, Kunkel A, Vanderborght J, Javaux M, Oberdörster C, Hammel K, Schnepf A (2021). Mechanistic modeling of pesticide uptake with a 3D plant architecture model. Environ Sci Pollut Res.

[CR32] Juraske R, Mosquera-Vivas CS, Erazo-Velásquez A, García-Santos G, Berdugo-Moreno MB, Díaz-Gómez J, Binder CR, Hellweg S, Guerrero-Dallos JA (2011). Pesticide uptake in potatoes: model and field experiments. Environ Sci Technol.

[CR33] Kah M, Beulke S, Brown CD (2007). Factors influencing degradation of pesticides in soil. J Agric Food Chem.

[CR34] Kaur P, Bhatia A, Kaur H, Bhullar MS (2023). Adsorption and desorption of Imazethapyr in Indian soils in relation to soil properties and temperature. Int J Environ Sci Technol.

[CR35] Keinan A, Clark AG (2012). Recent explosive human population growth has resulted in an excess of rare genetic variants. Science.

[CR36] Khan MA, Brown CD (2016). Influence of commercial formulation on leaching of four pesticides through soil. Sci Total Environ.

[CR37] Khandelwal A, Narayanan N, Varghese E, Gupta S (2020). Linear and nonlinear isotherm models and error analysis for the sorption of kresoxim-methyl in agricultural soils of India. Bull Environ Contam Toxicol.

[CR38] Laabs V, Amelung W, Pinto A, Zech W (2002). Fate of pesticides in tropical soils of Brazil under field conditions. J Environ Qual.

[CR39] Lammoglia S-K, Brun F, Quemar T, Moeys J, Barriuso E, Gabrielle B, Mamy L (2019). Modelling pesticides leaching in cropping systems: effect of uncertainties in climate, agricultural practices, soil and pesticide properties. Environ Modell Softw.

[CR40] Langeron J, Blondel A, Sayen S, Hénon E, Couderchet M, Guillon E (2014). Molecular properties affecting the adsorption coefficient of pesticides from various chemical families. Environ Sci Pollut Res.

[CR41] Liang B, Yang C, Gong M, Zhao Y, Zhang J, Zho C, Jiang J, Li S (2011). Adsorption and degradation of triazophos, chlorpyrifos and their main hydrolytic metabolites in paddy soil from Chaohu Lake. China J Environ Manage.

[CR42] Liang H, Li L, Li W, Wu Y, Zhou Z, Liu F (2011). Dissipation and residue of dimethomorph in pepper and soil under field conditions. Ecotox Environ Safe.

[CR43] Liu C, Wan K, Huang J, Wang Y, Wang F (2012). Behavior of mixed formulation of metalaxyl and dimethomorph in grape and soil under field conditions. Ecotox Environ Safe.

[CR44] López-Piñeiro A, Sñanchez-Terrón J, Martín-Franco C, Peña D, Vicente LA, Gómez S, Fernández-Rodríguez D, Albarrán A (2022). Impacts of fresh and aged holm-oak biochar on clomazone behaviour in rice cropping soils after transition to sprinkler irrigation. Geoderma.

[CR45] Ma CS, Zhang W, Peng Y, Zhao F, Chang X-Q, Xing K, Zhu L, Ma G, Yang H-P, Rudolf VHW (2021). Climate warming promotes pesticide resistance through expanding overwintering range of a global pest. Nat Commun.

[CR46] Maillard E, Payraudeau S, Faivre E, Grégoire C, Gangloff S, Imfeld G (2011). Removal of pesticides mixtures in a storm water wetland collecting runoff from a vineyard catchment. Sci Total Environ.

[CR47] Mamy L, Barriuso E (2007). Desorption and time-dependent sorption of herbicides in soils. Eur J Soil Sci.

[CR48] McGrath G, Rao PSC, Mellander P-E, Kennedy I, Roseg M, van Zwieten L (2019). Real-time forecasting of pesticide concentrations in soil. Sci Total Environ.

[CR49] Mojica AP, Guerrero-Dallos JA (2013). Evaluation of pesticide movement towards Tota lake catchment. Colombia Rev Colom Quim.

[CR50] Mosquera-Vivas CS, Hansen EW, García-Santos G, Obregón-Neira N, Celis-Ossa RE, González-Murillo CA, Juraske R, Hellweg S, Guerrero-Dallos JA (2016). The effect of the soil properties on adsorption, single-point desorption, and degradation of chlorpyrifos in two agricultural soil profiles from Colombia. Soil Sci.

[CR51] Mosquera-Vivas CS, Martínez-Cordón MJ, García-Santos G, Guerrero-Dallos JA (2017). Temporal distribution of pyrimethanil and dimethomorph fungicides on an Andisol under cut rose production in Colombia. Höhere Bundeslehr-und Forschungsanstalt für Landwirtschaft Raumberg-Gumpenstein. 17. Gumpensteiner Lysimetertagung.

[CR52] Mosquera-Vivas CS, Martínez MJ, García-Santos G, Guerrero-Dallos JA (2018). Adsorption-desorption and hysteresis phenomenon of tebuconazole in Colombian agricultural soils: experimental assays and mathematical approaches. Chemosphere.

[CR53] Mosquera-Vivas CS, Obregón-Neira N, Celis-Ossa RE, Guerrero-Dallos JA, González-Murillo CA (2016). Degradation and thermodynamic adsorption process of carbofuran and oxadicyl in a Colombian agricultural soil profile. Agron Colomb.

[CR54] Mottes C, Lesueur-Jannoyer M, Charlier J-B, Carles C, Guéné M, Le Bail M, Malézieux E (2015). Hydrological and pesticide transfer modeling in a tropical volcanic watershed with the WATPPASS model. J Hydrol.

[CR55] Mukherjee S, Tappe W, Weihermueller L, Hofmann D, Köppchen S, Laabs V, Schroeder T, Vereecken H, Burauel P (2016). Dissipation of bentazone, pyrimethanil and boscalid in biochar and digestate based soil mixtures for biopurification systems. Sci Total Environ.

[CR56] Ngan CK, Cheah UB, Wan-Abdullah WY, Lim KP, Ismail BS (2005). Fate of chlorothalonil, chlorpyrifos and profenofos in vegetable farm in Cameron Highlands. Malaysia Water Air Soil Poll.

[CR57] Ogram AV, Jessup RE, Ou LT, RAO PSC, (1984). Effects of sorption on biological degradation rates of (2,4-Dichlorophenoxy) acetic acid in soils. Appl Environ Microbiol.

[CR58] Oltramare C, Weiss FT, Staudacher P, Kibirango O, Atuhaire A, Stamm C (2023). Pesticides monitoring in surface water of a subsistence agricultural catchment in Uganda using passive samplers. Environ Sci Pollut Res.

[CR59] Papadopoulou ES, Karas PA, Nikolaki S, Storck V, Ferrari F, Trevisan M, Tsiamis G, Martin-Laurent F, Karpouzas DG (2016). Dissipation and adsorption of isoproturon, tebuconazole, chlorpyrifos and their main transformation products under laboratory and field conditions. Sci Total Environ.

[CR60] Paraiba L, Spadotto C (2002). Soil temperature effect in calculating attenuation and retardation factors. Chemosphere.

[CR61] Park WP, Chang KM, Hyun HN, Boo K-H, Koo B-J (2020). Sorption and leaching characteristics of pesticides in volcanic ash soils of Jeju Island. Korea. Appl Biol Chem.

[CR62] Pathak VM, Verma VK, Rawat BS, Kaur B, Babu N, Sharma A, Dewali S, Yadav M, Kumari R, Singh S, Mohapatra A, Pandey VR, Cunill JM (2022). Current status of pesticide effects on environment, human health and it’s eco-friendly management as bioremediation: a comprehensive review. Front Microbiol.

[CR63] Piao XY, Tao CJ, Jiang H, Wang XJ (2011). Study on degradation dynamics of Z-and E-isomers of dimethomorph in soils Chinese J. Pest Sci.

[CR64] PPDB (Pesticide Properties DataBase). Available at http://sitem.herts.ac.uk/aeru/ppdb/en/. Accessed 12 2022.

[CR66] Ramakrishnan B, Venkateswarlu K, Sethunathan N, Megharaj M (2019). Local applications but global implications: Can pesticides drive microorganisms to develop antimicrobial resistance?. Sci Total Environ.

[CR67] Rao PSC, Hornsby AG, Jessup RE (1985). Indices for ranking the potential for pesticide contamination of groundwater. Proc Soil Crop Sci Soc Fla.

[CR68] Redondo MJ, Ruiz MJ, Font G, Boluda R (1997). Dissipation and distribution of atrazine, simazine, chlorpyrifos and tetradifon residues in citrus orchard soils. Arch Environ Contam Toxicol.

[CR69] Rojas R, Morillo J, Usero J, Delgado-Moreno L, Gan J (2013). Enhancing soil sorption capacity of an agricultural soil by addition of three different organic wastes. Sci Total Environ.

[CR70] Saha A, Ghosh RK, Bhaduri D, Rakshit A, Singh S, Abhilash P, Biswas A (2021). Pesticide pollution in soils and sediment in india: status, impact and countermeasures. Soil science: fundamentals to recent advances.

[CR71] Shrestha S, Datta A (2015). Field measurements for evaluating the RZWQM and PESTFADE models for the tropical zone of Thailand. J Environ Manage.

[CR72] Šimůnek J, van Genuchten MT (2008). Modeling nonequilibrium flow and transport processes using HYDRUS. Vadose Zone J.

[CR73] Singh RP, Srivastava G (2009). Adsorption and movement of carbofuran in four different soils varying in physical and chemical properties. Adsorpt Sci Technol.

[CR74] Srimurali S, Govindaraj S, Krishna Kumar S, Babu Rajendran R (2015). Distribution of organochlorine pesticides in atmospheric air of Tamilnadu, southern India. Int J Environ Sci Technol.

[CR75] Valderde-García A, Socías-Viciana M, González-Pradas E, Villafranca-Sánchez M (1992). Adsorption of chlorpyrifos on Almería soils. Sci Total Environ.

[CR76] Vanni A, Anfossi L, Cignetti A, Baglieri A, Gennari M (2006). Degradation of pyrimethanil in soil: influence of light, oxygen, and microbial activity. J Environ Sci Health B.

[CR77] Wang C, Zhang Q, Wang F, Liang W (2017). Toxicological effects of dimethomorph on soil enzymatic activity and soil earthworm (*Eisenia fetida*). Chemosphere.

[CR78] Wolt J, Singh P, Cryer S, Lin J (2009). Sensitivity analysis for validating expert opinion as to ideal data set criteria for transport modeling. Environ Toxicol Chem.

[CR79] Yu YL, Wu XM, Li SN, Fang H, Zhan HY, Yu JQ (2006). An exploration of the relationship between adsorption and bioavailability of pesticides in soil to earthworm. Environ Pollut.

[CR80] Yu X, Pan L, Ying G, Kookana RS (2010). Enhanced and irreversible sorption of pesticide pyrimethanil by soil amended with biochars. J Environ Sci.

[CR81] Zaller JG, Kruse-Plaß M, Schlechtriemen U, Gruber E, Peer M, Nadeem I, Formayer H, Hutter H-P, Landler L (2022). Pesticides in ambient air, influenced by surrounding land use and weather, pose a potential threat to biodiversity and humans. Sci Total Environ.

[CR82] Zhang C, Li J, Wu X, Long Y, An H, Pan X, Li M, Dong F, Zheng Y (2022). Rapid degradation of dimethomorph in polluted water and soil by Bacillus cereus WL08 immobilized on bamboo charcoal–sodium alginate. J Hazard Mater.

[CR83] Zhou Y, Jing J, Yu R, Zhao Y, Gou Y, Tang H, Zhang H, Huang Y (2023). Distribution of pesticide residues in agricultural topsoil of the Huangshui catchment, Qinghai Tibet Plateau. Environ Sci Pollut Res.

