# Supplementary material for: Empirical model to assess leaching of pesticides in soil under a steady-state flow and tropical conditions
Source: Int J Environ Sci Technol (Tehran). 2023 Aug 20;21(2):1301–20. doi: 10.1007/s13762-023-05038-w (PMC10784402; doi:10.1007/s13762-023-05038-w)
Supplement: Supplementary file 1 — Supplementary file1 (DOCX 5396 KB) [file 13762_2023_5038_MOESM1_ESM.docx]

**Supporting Information of the EMOLP model**

**1. Multicriteria Analysis (MCA)**

The selection of the pesticides studied in the laboratory (adsorption-desorption and degradation processes) and field assays were based on a Multicriterion Analysis (MCA) Score. The MCA took into account the following steps:

a) **general goal of the problem**: to select two pesticides for study

b) **alternatives**: the pesticides applied during 2010

c) **criteria**: summarized in Table S1

d) **weighting**: the weighting was equal among all criteria

e) **score of the criteria**: Table S2 shows the assigned score for each criterion

f) **calculation of the final score**: calculated using Eq. (S1):

$S_{j}= \sum_{i} p_{i} C_{ij}$ (Eq.S1)

where $S_{j}$ is the final score, $p_{i}$ is the weighting of each criterion (equal to one) and $C_{ij}$ is the score for criterion $j$.

Table S1. Criteria used in the Multicriterion Analysis to select pesticides for study

| Physicochemical characteristics  of the pesticides | Criterion |
| --- | --- |
| Solubility in water (*S_w_*) at 20 ºC | High, when *S_w_* > 100 mg L^-1^ |
| Volatility (*K_H_*) using Henry’s Law at 25 ºC | Low, when *K_H_* < 3.0 E-07 atm m^3^ mol^-1^ |
| Adsorption coefficient (*Log K_oc_*) and half-life (*t_1/2_*) using de GUS Index | High, when GUS Index > 2.8 |
| Dose applied (*D_app_*) | High, when *D_app_* > 1.1 cc o g L^-1^ |
| Frequency of application (*F_app_*) | Very high, when *F_app_* > 53 applications |
| *LD_50_* of ingestion in mammals | High, when *LD_50_* < 5 mg kg^-1^ |
| Hydrolysis (*H*) at pH = 7.0 and 20 ºC | Low, when *H* > 180 d |
| Application method (*W_app_*) | High, when *W_app_* = soil application |

Table S2. Score and classification of each criterion

| \| Solubility a 20 ºC (mg L^-1^) \| Classification \| Score \| \| --- \| --- \| --- \| \| < 0.1 \| Not soluble \| **10** \| \| 0.1 – 1.0 \| Slightly soluble \| **20** \| \| 1.0 – 10 \| Moderately soluble \| **30** \| \| 10 – 100 \| Easily soluble \| **40** \| \| > 100 \| Highly soluble \| **50** \| |
| --- | --- | --- | --- | --- | --- | --- | --- | --- | --- | --- | --- | --- | --- | --- | --- | --- | --- | --- |
| \| Henry’s Law constant at 25ºC (atm m^3^ mol^-1^) \| Classification \| Score \| \| --- \| --- \| --- \| \| < 3.0 E^-07^ \| Not volatile \| **40** \| \| 3.0 E^-07^ – 1.0 E^-05^ \| Low volatility \| **30** \| \| 1.0 E^-05^ – 1.0 E^-03^ \| Moderate volatility \| **20** \| \| > 1.0x10^-03^ \| High volatility \| **10** \| |
| \| **GUS Index** \| **Classification** \| **Score** \| \| --- \| --- \| --- \| \| < 1.8 \| Non-leacher \| **10** \| \| 1.8 – 2.8 \| Bordeline \| **50** \| \| > 2.8 \| Leacher \| **100** \| |
| \| **Dose range (cc o g L^-1^)** \| **Score** \| \| --- \| --- \| \| < 0.1 \| **10** \| \| 0.1 – 0.3 \| **20** \| \| 0.3 – 0.5 \| **30** \| \| 0.5 – 0.7 \| **40** \| \| 0.7 – 0.9 \| **50** \| \| 0.9 – 1.1 \| **60** \| \| > 1.1 \| **70** \| |
| \| **Frequency of application** \| **Classification** \| **Score** \| \| --- \| --- \| --- \| \| 1 – 2 \| Very low \| **10** \| \| 3 – 6 \| Low \| **20** \| \| 7 – 12 \| Slightly Moderate \| **30** \| \| 13 – 25 \| Moderate \| **40** \| \| 26 – 52 \| High \| **50** \| \| 53 – 104 \| Very high \| **60** \| |
| \| LD_50_ (mg kg^-1^) \| Classification \| Score \| \| --- \| --- \| --- \| \| < 5 \| Extremely dangerous \| **40** \| \| 5 – 50 \| Highly dangerous \| **30** \| \| 50 – 500 \| Moderately dangerous \| **20** \| \| > 500 \| Slightly dangerous \| **10** \| |
| \| **DT_50_ at pH=7.0 and 20ºC (days)** \| **Classification** \| **Score** \| \| --- \| --- \| --- \| \| < 20 \| Easily degradable \| **10** \| \| 20 – 60 \| Somewhat degradable \| **20** \| \| 60 – 180 \| Slightly degradable \| **30** \| \| > 180 \| Very slightly degradable \| **40** \| \| Stable  (360 d) \| Stable \| **50** \| |
| \| **Application method** \| **Classification** \| **Score** \| \| --- \| --- \| --- \| \| Foliage \| Low \| **10** \| \| Drench- soil \| High \| **100** \| |

The data and classifications of solubility, volatility, adsorption coefficient, half-life, 50% lethal dose by ingestion by mammals and hydrolysis of the pesticides were obtained from the Footprint and FAO websites. The data on dose applied, frequency of application and application method were collected in the field. Table S3 summarizes the results of the MCA of the 27 pesticides applied during 2010. Dimethomorph and pyrimethanil were the two fungicides that had the highest scores and were thus selected for the field and laboratory studies.

Table S3. Results of the Multicriterion Analysis

| **Active ingredient** | **Disease** | **Total Score** |
| --- | --- | --- |
| Dimethomorph | Downy mildew | **340** |
| Pyrimethanil | Botrytis | **310** |
| Clothianidin | Thrips-aphids | **300** |
| Thiamethoxam | Thrips-aphids | **290** |
| Propamocarb hydrochloride | Downy mildew | **280** |
| Boscalid | Botrytis | **260** |
| Iprodione | Botrytis | **260** |
| Methomyl | Thrips-aphids | **260** |
| Acetamiprid | Thrips-aphids | **250** |
| Oxycarboxin | Blight | **250** |
| Domemorph acetate | Powdery mildew | **240** |
| Carbendazim | Botrytis | **240** |
| Thiocyclam hidrogen oxalate | Thrips-aphids | **240** |
| Mandipropamid | Downy mildew | **230** |
| Prochloraz | Botrytis | **230** |
| Acephate | Thrips-aphids | **220** |
| Triflumizole | Powdery mildew | **220** |
| Propamocarb | Downy mildew | **210** |
| Spinosad | Thrips-aphids | **210** |
| Spiroxamine | Powdery mildew | **210** |
| Difenoconazole | Powdery mildew | **200** |
| Penconazole | Powdery mildew | **200** |
| Pyraclostrobin | Powdery mildew | **180** |
| Pyriproxyfen | Thrips-aphids | **180** |
| Fenamidone | Downy mildew | **170** |
| Malathion | Thrips-aphids | **160** |
| Metrafenone | Powdery mildew | **160** |

**2. Conceptual model of the Empirical Model of Leaching of Pesticides EMOLP**

The conceptual model of the EMOLP is shown in Fig. S1.


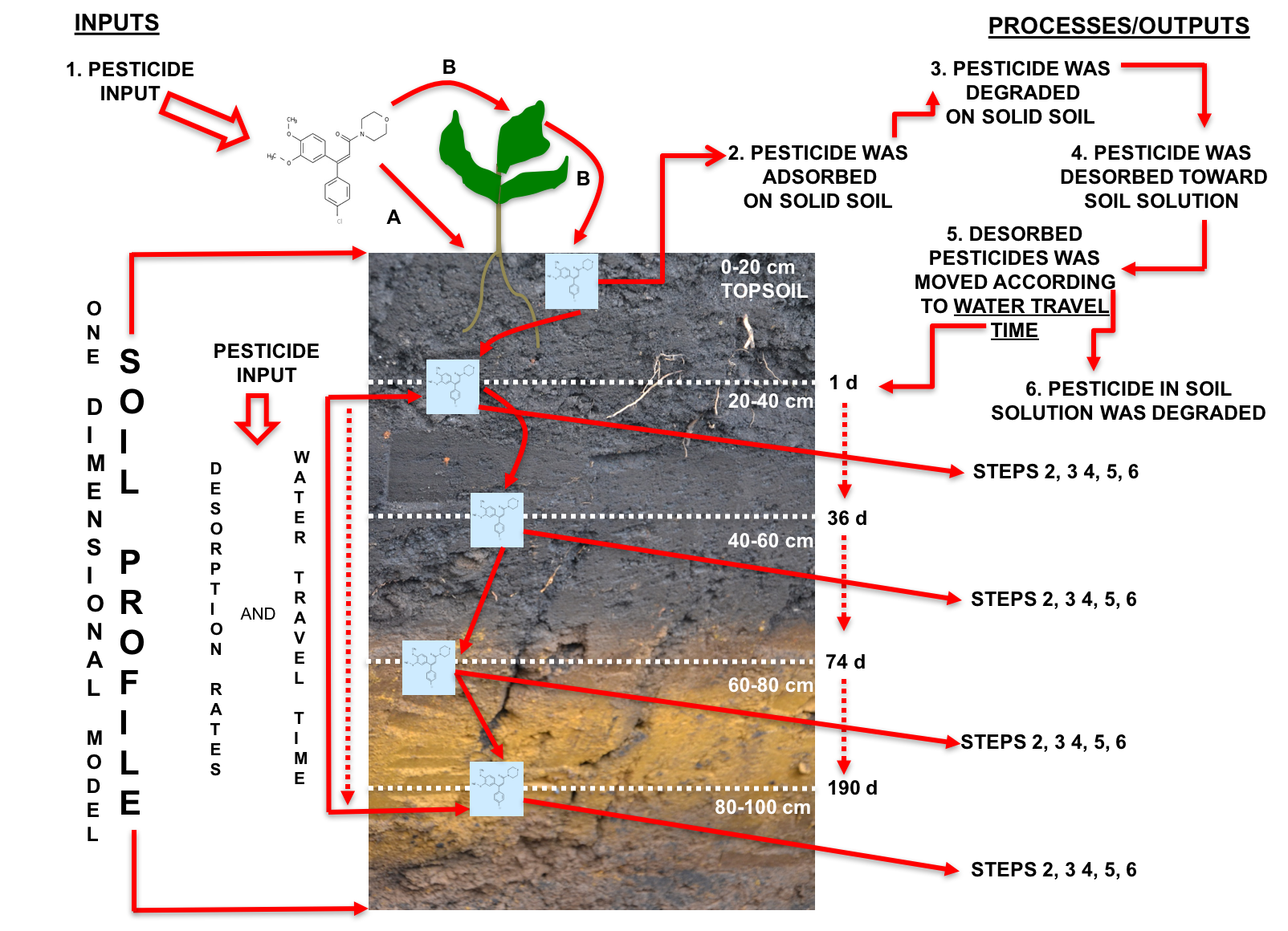


Fig. S1. Conceptual model of the EMOLP

The values of adsorption coefficients and percentages of dimethomorph and pyrimethanil with two different initial concentration ranges are summarized in Table S4.

Table S4. Adsorption coefficients and percentages with different initial concentration ranges

| Pesticide | Soil type | Soil layer - depth  (cm) | Linear model  Initial concentration range I | | | Linear model  Initial concentration range II | | |
| --- | --- | --- | --- | --- | --- | --- | --- | --- |
|  |  |  |  | |  |  | | |
|  |  |  | $\boldsymbol{K}_{\boldsymbol{d}}$**, L^-1^ kg** | **R^2^** | Percentage of adsorption,  %ads | $\boldsymbol{K}_{\boldsymbol{d}}$**, L^-1^ kg** | **R^2^** | Percentage of adsorption,  %ads |
| Pyrimethanil | Andisol | SL1 (0-20)  SL2 (20-40)  SL3 (40-60)  SL4 (60-80)  SL5 (80-100) | 119.1 ± 6.4  170.4 ± 9.2  171.8 ± 7.6  31.5 ± 1.5  8.1 ± 0.6 | 0.98  0.98  0.98  0.98  0.96 | 98.8 ± 0.5  99.1 ± 0.4  99.1 ± 0.4  95.5 ± 1.4  84.5 ± 3.5 | 251.0 ± 27.8  369.0 ± 34.8  360.7 ± 28.4  52.48 ± 6.1  9.26 ± 1.3 | 0.97  0.97  0.98  0.96  0.94 | 99.3 ± 0.1  99.6 ± 0.1  99.5 ± 0.1  97.1 ± 0.7  85.8 ± 3.1 |
| Dimethomorph | Andisol | SL1 (0-20)  SL2 (20-40)  SL3 (40-60)  SL4 (60-80)  SL5 (80-100) | 35.3 ± 1.9  27.1 ± 0.8  17.1 ± 1.5  7.0 ± 0.5  4.5 ± 0.1 | 0.98  0.99  0.94  0.96  0.99 | 93.3 ± 1.7  93.0 ± 2.0  90.3 ± 2.3  78.6 ± 4.1  67.5 ± 1.7 | 94.8 ± 4.0  75.3 ± 4.0  47.4 ± 3.5  17.8 ± 1.0  7.7 ± 0.3 | 0.99  0.99  0.98  0.98  0.99 | 97.0 ± 0.5  96.9 ± 0.7  94.2 ± 0.8  85..3 ± 2.4  72.0 ± 7.1 |
| Pyrimethanil: initial concentration range I = 0.6 - 9.8 mg L^-1^ and initial concentration range II = 0.6 - 2.1 mg L^-1^  Dimethomorph: initial concentration range I = 0.3 - 5.4 mg L^-1^ and initial concentration range II = 0.3 - 1.6 mg L^-1^ | | | | | | | | |

**3. Thermodynamic approach of the adsorption process**

The partial molar free energy of adsorption,${\Delta G}_{ads}^{^{\circ}}$, at the reference temperature ($T_{r}$ = 20 °C) was calculated from Eq. (S2) (Mosquera-Vivas et al., 2016a):

${\Delta G}_{ads}^{^{\circ}}= -RTlnK_{d}$ (Eq. S2)

where $R$ is the gas constant and $K_{d}$ is the distribution coefficient of the fungicide between the liquid and solid soil phases.

The enthalpy of adsorption (${\Delta H}_{ads}$) was calculated according to Eq. (S3) and Eq. (S4):

${\Delta H}_{ads}= -4.17ln\left( {K_{d}^{T_{r}}}/{1.0 x {10}^{3} \bar{SS}} \right)-88.1$ (Eq. S3)

$\bar{SS}=100.0 \left( 100.0f_{oc}+2.0f_{cl}+0.4f_{st}+0.005f_{sd} \right)$ (Eq. S4)

where $K_{d}^{T_{r}}$ is the coefficient of distribution at $T_{r}$ and $\bar{SS}$ is the average specific surface of the soil. $f_{oc}$*,*$f_{cl}$*,* $f_{st}$ and $f_{sd}$ represent the fractions of carbon, clay, silt and sand in the soil (Gross and Schwarzenbach, 1999; Vighi and Di Guardo, 1995).

**4. The effect of soil pH on pesticide adsorption**

Determining whether a pesticide was protonated or deprotonated allowed us to predict whether the pesticide would be retained in the soil. For instance, an acidic pesticide will be deprotonated when the soil pH is higher than the acid constant of the substance $pK_{a}$; therefore, the negative net of the soil repels the compound. The deprotonated and protonated concentration of acidic and basic pesticides were calculated using Eq. (S5) and Eq. (S6):

$\left[ A^{-} \right]=\frac{\left[ HA \right]}{\left( \frac{{10}^{-pH}}{{10}^{-pK_{a}}}+1 \right)}$ (Eq. S5)

$\left[ B^{+} \right]= \frac{\left[ B \right]}{\left( \frac{{10}^{-pK_{a}}}{{10}^{-pH}}+1 \right)}$ (Eq. S6)

where $\left[ A^{-} \right]$ is the concentration (mole L^-1^) of the deprotonated acidic pesticide, $\left[ HA \right]$ is the total concentration (mole L^-1^) of the acidic pesticide, $pH$ is the concentration (mole L^-1^) of $H^{+}$ in the soil, $B^{+}$ is the concentration (mole L^-1^) of the protonated basic pesticide and $\left[ B \right]$ is the total concentration (mole L^-1^) of the basic pesticide.

**5. Pesticide deposition on soil surface during foliar applications**

The fungicides dimethomorph and pyrimethanil were used in foliar applications in the study plot during the field trials. The fungicides deposited on the soil after foliar application depends on the following factors: stage of the crop, the crop type, application method, type of spray equipment and meteorological conditions (García-Santos et al., 2011). In order to calculate the percentage of dimethomorph and pyrimethanil that reaches the soil surface after spray application, the greenhouse (study plot) was split into two sub-plots (U1 and U2) at different stages of cut rose cultivation. The height of the roses in sub-plot U1 was between 0.10 and 0.60 m, while the height of the flowers in sub-plot U2 varied between 0.30 and 1.75 m. Six Petri Dishes lined with paper were placed on the soil of several furrows of the U1 and U2 sub-plots (Fig. S2). The dishes were located between plants in the middle of the furrows every 5.88 m ± 0.38 m in a Zig-Zag pattern. Two hours after spray application, the samples were collected, extracted with ethyl acetate and shaken for 30 min. The organic phase was concentrated to 2 mL and an aliquot of PCB 52 (Internal Standard) was added to the samples before injection into the CG-MS system. The amounts of the fungicides were related to unit of paper area, and the percentage of fungicide deposited on soils was calculated as:

$\% P_{D}= \frac{\left( \frac{P_{E}}{A_{PB}} \right)}{P_{A}} x 100\%$ (Eq. S7)

where $\% P_{D}$ is the percentage of the fungicide deposited on soil, $P_{E}$ is the amount of the extracted pesticide (mg), $A_{PB}$ is the area of Petri Dish (m^2^) and $P_{A}$ is the amount of pesticide applied in the furrow (mg).

In addition, a drift assay was carried out in sub-plot U1. Six paper-lined Petri dishes were placed in the furrow where the plant was sprayed and 0.65 m, 1.3 m, 2.6 m away from the plant (Fig. S2). The pesticide was quantified as above.

**
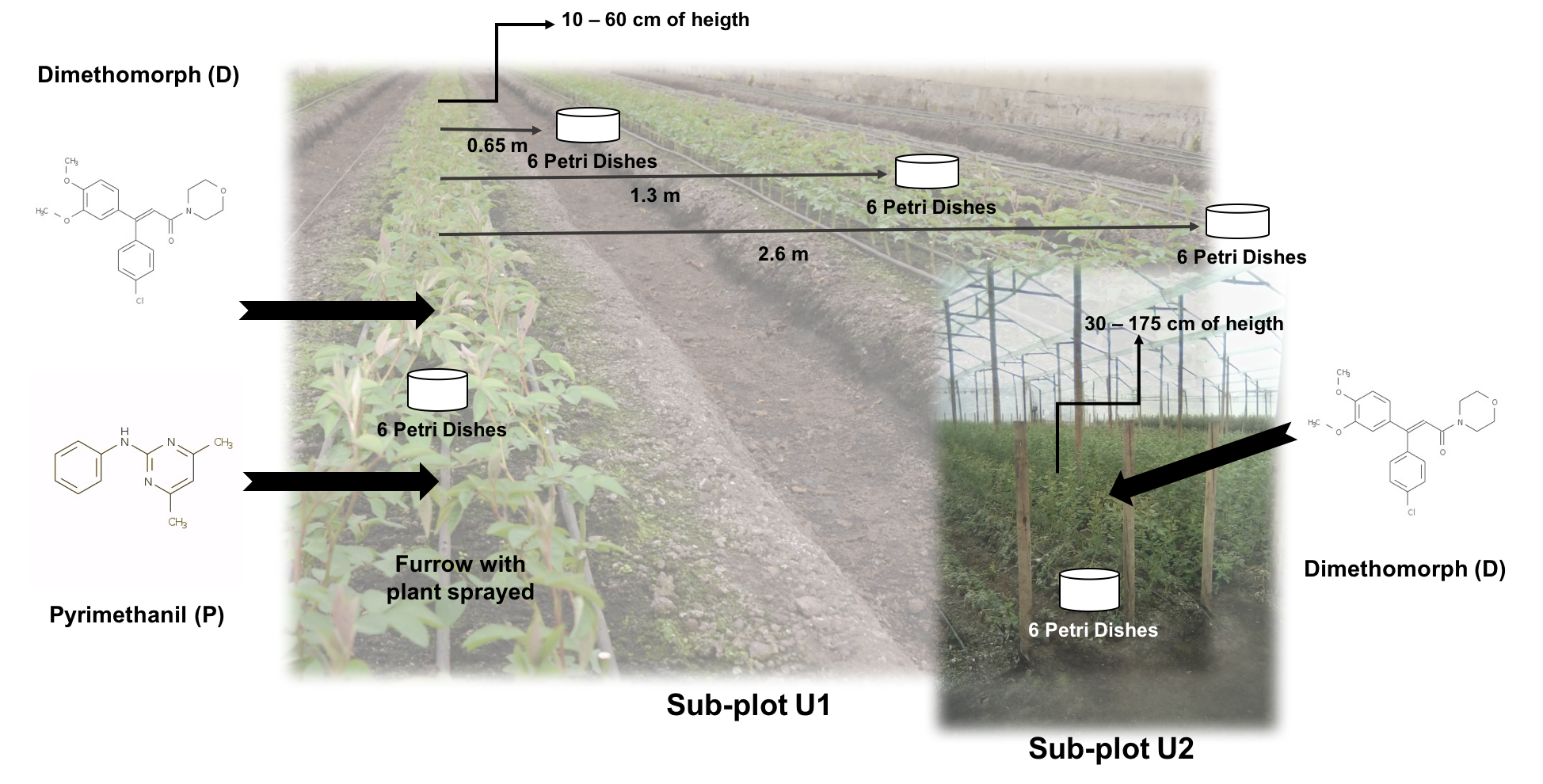
**

Fig. S2. Assay of the fraction of fungicide deposited on topsoil after foliar application.

**6. Fungicide applications during the field trials**

Table S5 shows the dates and doses of the fungicides dimethomorph and pyrimethanil, as well as bromide (conservative tracer) applied during the field trials in T-1 and T-2.

Table S5. Applications of the fungicides and bromide during the field trials

| Pesticide or compound | Date of application | Doses of active ingredient, kg ha^-1^ |  | Application mode |  |
| --- | --- | --- | --- | --- | --- |
| Dimethomorph | 19-May-12  24-May-12  31-May-12  28-Jun-12  6-Sep-12  22-Mar-13  10-May-13  7-Jun-13  12-Jun-13  19-Jun-13  3-Jul-13  6-Jul-13  12-Jul-13 | 12.10  0.64  0.64  0.64  12.10  0.80  0.72  0.80  0.64  0.64  0.57  0.80  0.80 |  | Soil (T-1)  Foliage (T-1)  Foliage (T-1)  Foliage (T-1)  Soil (T-2)  Foliage (T-1 and T-2)  Foliage (T-1 and T-2)  Foliage (T-2)  Foliage (T-2)  Foliage (T-2)  Foliage (T-2)  Foliage (T-2)  Foliage (T-2) |  |
| Pyrimethanil | 6-Sep-12  14-Sep-12  18-Sep-12  2-Oct-12  25-Dec-12  23-Feb-13  27-Mar-13  27-Apr-13  17-May-13  22-May-13  14-Jun-13  19-Jun-13  21-Aug-13 | 20.16  1.17  1.17  0.73  1.17  1.17  1.46  1.46  1.46  1.46  1.17  1.17  1.46 |  | Soil (T-2)  Foliage (T-2)  Foliage (T-2)  Foliage (T-2)  Foliage (T-2)  Foliage (T-2)  Foliage (T-2)  Foliage (T-2)  Foliage (T-2)  Foliage (T-2)  Foliage (T-2)  Foliage (T-2)  Foliage (T-2) |  |
| Bromide | 19-May-12  6-Sep-12 | 22.33  123.77 |  | Soil (T-1)  Soil (T-2) |  |
| T-1: first field trial; T-2: second field trial | | | | | |

**7. Travel time of a non-reaction substance**

The travel time of a non-reaction substance was calculated according to Rao et al. 1985 (Eq. (S8)):

$t_{d}= {L \theta_{fc} RF}/{J_{w}}$ (Eq. S8)

where $t_{d}$ is the travel time of a non-reaction substance, $L$ is the soil depth (m), $\theta_{fc}$ is the soil water volumetric content at field capacity, $RF$ is the retardation factor, and $J_{w}$ is the soil average daily water net recharge (m day^-1^). For the non-reaction substance or conservative tracer (bromide), $RF$ is equal to one; therefore, ${L \theta_{fc}}/{J_{w}}$ estimates water flow travel time or bromide (non-reaction compound) travel time. The travel time of the conservative tracer between the first (0-20 cm) and the second (20-40 cm) soil layers was equal to 38 days with $L$ = 0.20 m, $\theta_{fc}$ = 0.38 and $J_{w}$ = 2.03 x 10^-03^ m d^-1^. The soil average daily water net recharge was calculated using a daily input of water equal to 4.03 mm and a daily output of water equal to 2.0 mm.

During the field trials, the travel time of the bromide between the first (0-20 cm) and the second (20-40 cm) soil layer was equal to 1 day; we collected an undisturbed soil column in the greenhouse with an acrylic tube 30 cm in diameter and 100 cm in length to obtain the arrival time of the conservative tracer to depths of 20 cm, 40 cm, 60 cm, and 80 cm under controlled conditions (Fig. S3). It was transported to the laboratory, and four effluent sampling points were located throughout the length of the soil column at heights of 20 cm, 40 cm, 60 cm and 80 cm. Two porous capsules were installed on opposite sides of the acrylic tube at the same heights as the sampling points. A 50 cm x 50 cm square base supported the acrylic tube and had a final outlet sampling point. The undisturbed soil column was held at an average temperature of 17.0 °C ± 2.0 °C and average relative humidity of 66.0 % ± 9.0 %. The daily soil temperature ranged from 12 °C to 24 °C at 10 cm depth, the water content at depth of 50 cm was measured with a TDR ThetaProbe ML2x/d, AT Delta-T Devices and varied from 0.392 m^3^ m^-3^ to 0.419 m^3^ m^-3^ (Mosquera-Vivas et al., 2017). Fig. S4 shows the travel time of bromide in the undisturbed soil column. It was detected 1d, 36 d, 74 d and 190 d after application at depths of 20 cm, 40 cm, 60cm and 80 cm, respectively.


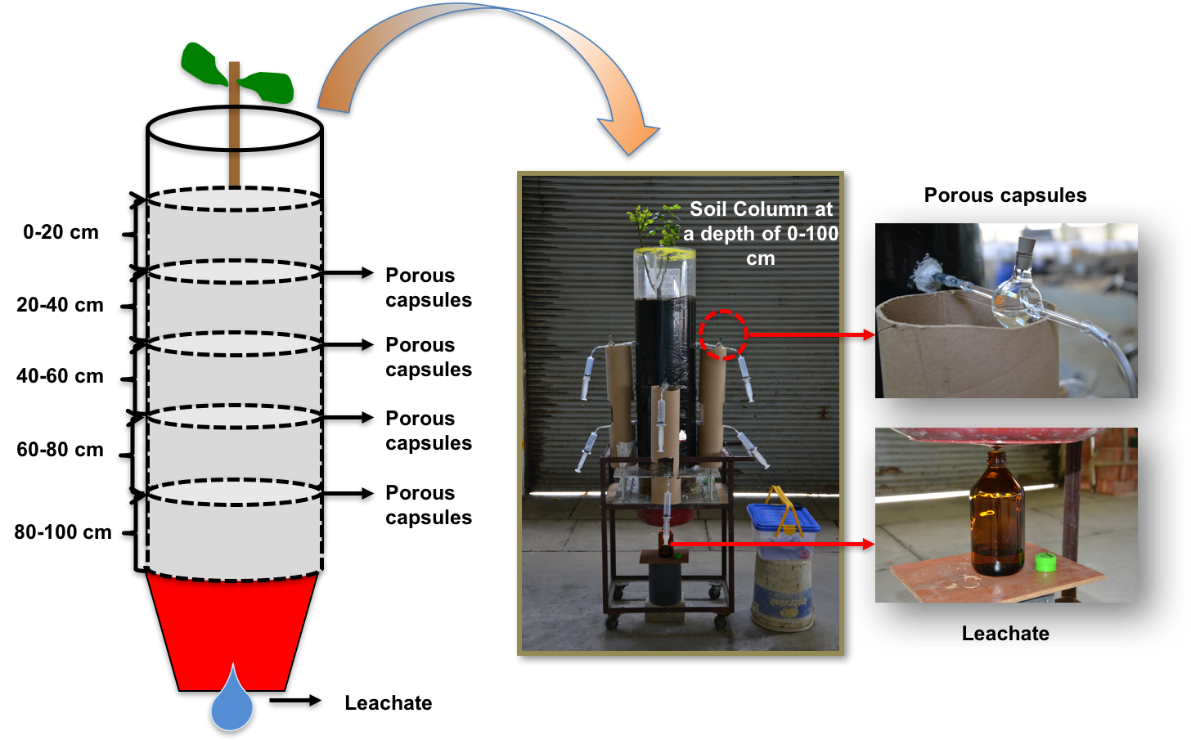


Fig. S3. Schematic diagram of the experimental undisturbed soil column


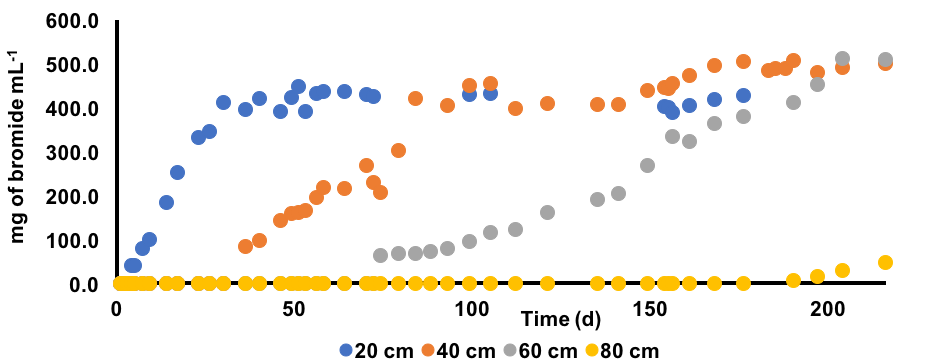


Fig. S4. Water flow travel time based on the travel time of the conservative tracer, bromide.

**8. Penetration depth during application of fungicides directly on the soil**

The behavior of dimethomorph, pyrimethanil and bromide (conservative tracer) over time in the two undisturbed soil columns at a depth of 0-20 cm are summarized in Fig. S5. The bromide was detected in both columns at a depth of 12 cm and 20 cm; meanwhile, the fungicides were only detected in the column 2 at a depth of 12 cm. Bromide breakthrough curves at a depth of 20 cm were described by the convection-dispersion equation (CDE):

$\frac{\partial C}{\partial t}=D\frac{\partial^{2}C}{{\partial x}^{2}}- \nu\frac{\partial C}{\partial x}$ (Eq. S9)

where $\frac{\partial C}{\partial t}$ is the change of the concentration of bromide over time, $\frac{\partial C}{\partial x}$ is the change of the concentration of bromide in the distance, $\nu$ is the average pore-water velocity (L/T) and $D$ is the dispersion coefficient (L^2^/T). The values of $\nu$ and $D$ were calculated using the software STANMOD 2.0 with CXFITM code in equilibrium and non-equilibrium (Šimůnek et al., 1999). The Péclet number $P_{e}$ (Eq. S10) determines whether transport by advection should be considered; when the Péclet number is smaller than 1, diffusion is considered to be the dominant transport mechanism of the solutes in the soil (Huysmans & Dassargues, 2004).

$P_{e}= \frac{\nu}{D} \times L$ (Eq. S10)

where $L$ is the lenght of the column.

The transport parameters of the soil described with CDE in equilibrium and non-equilibrium and the Péclet number are shown in Table S6.

Table S6. Transport parameters of the soil in equilibrium and non-equilibrium at a depth of 0-20 cm

|  | **Equilibrium** |  |  |  |  | **Non-equilibrium** |  |  |  |  |  |  |
| --- | --- | --- | --- | --- | --- | --- | --- | --- | --- | --- | --- | --- |
| **Column** | $\boldsymbol{\nu}$  **cm d^-1^** | $\boldsymbol{D}$  **cm^2^ d^-1^** | **R^2^** | **MSE** | $P_{e}$ | $\boldsymbol{\nu}$  **cm/d** | $\boldsymbol{D}$  **cm^2^ d^-1^** | $\boldsymbol{\beta}$ | $\boldsymbol{\omega}$  **d^-1^** | **R^2^** | **MSE** | $P_{e}$ |
| 1 | 0,32  ± 0,008 | 1,73  ± 0,05 | 0,95 | 201 | 3,70 | 0,21  ± 0,06 | 0,81  ± 0,15 | 0,57  ± 0,13 | 0,46  ± 0,06 | 0,96 | 153 | 5,19 |
| 2 | 0,33  ± 0,009 | 2,32  ± 0,06 | 0,96 | 112 | 2,84 | 0,19  ± 0,007 | 0,90  ± 0,04 | 0,49  ± 0,02 | 0,57  ± 0,03 | 0,98 | 52 | 4,22 |
| R^2^: coefficient of determination, MSE: mean square error, $\beta$: partition coefficient between the mobile and immobile liquid phase and $\omega$: transfer of mass between the two zones (mobile and immobile). | | | | | | | | | | | | |

Fig. S5. Distribution of bromide, dimethomorph and pyrimethanil over time. A) Bromide breakthrough curves at a depth of 12 cm. Blue Points and line correspond to column 1, and red points and line correspond to column 2. B) Bromide breakthrough curve in column 1 at a depth of 20 cm. Blue points correspond to experimental data, orange line corresponds to simulation (CDE) in equilibrium and gray line corresponds to simulation (CDE) in non-equilibrium. C) Bromide breakthrough curve in column 2 at a depth of 20 cm. Blue points correspond to experimental data, orange line corresponds to simulation (CDE) in equilibrium and gray line corresponds to simulation (CDE) in non-equilibrium. D) Distribution of dimethomorph and pyrimethanil over time in column 2 at a depth of 12 cm. Blue points and line correspond to dimethomorph, and red points and line correspond to pyrimethanil. E) Degradation of dimethomorph in column 2 at a depth of 12 cm.

**9. Bromide quantification**

Bromide was quantified using an Agilent Technologies (Santa Clara, CA, USA) liquid chromatograph, model 1100, equipped with a UV detector and Allsep^™^ anion exchange column (150 mm x 4.6 mm x 7 μm). The mobile phase was 2.8 mM NaHCO_3_/ 2.2 mM Na_2_CO_3_ with a flow of 1.2 mL. Bromide retention time ranged from 4.3 to 5.4 min at 199 nm.

**10. Experimental concentration of the fungicides dimethomorph and pyrimethanil**

The experimental concentrations of the fungicides dimethomorph and pyrimethanil in T-1 and T-2 are shown in Table S7 and Table S8. Dimethomorph was applied once to the soil of T-1 and T-2 to calibrate and validate the empirical model, respectively. Pyrimethanil was only applied to the soil of T-2 to evaluate the mathematical equations.

Table S7. Experimental concentration of the fungicide dimethomorph in T-1 used to calibrate the empirical model

| Day, d | Soil layer depth, cm | | | | |
| --- | --- | --- | --- | --- | --- |
|  | **0-20** | **20-40** | **40-60** | **60-80** | **80-100** |
| 0 | 0.27 ± 0.04 | < LOD | < LOD | < LOD | < LOD |
| 1 | 17.86 ± 6.58 | 1.33 ± 0.29 | < LOD | < LOD | < LOD |
| 3 | 15.88 ± 4.56 | 0.64 ± 0.37 | < LOD | < LOD | < LOD |
| 7 | 28.49 ± 8.00 | 3.19 ± 1.23 | < LOD | < LOD | < LOD |
| 17 | 9.30 ± 3.10 | 1.11 ± 0.60 | < LOD | < LOD | < LOD |
| 28 | 9.09 ± 5.82 | 1.08 ± 0.78 | < LOD | < LOD | < LOD |
| 42 | 2.68 ± 0.11 | 0.06 ± 0.02 | < LOD | < LOD | < LOD |
| 63 | 0.56 ± 0.06 | 0.05 ± 0.03 | < LOD | < LOD | < LOD |
| 91 | 0.91 ± 0.14 | 0.06 ± 0.04 | < LOD | < LOD | < LOD |
| 141 | 0.63 ± 0.02 | 0.06 ± 0.02 | < LOD | < LOD | < LOD |
| 244 | 0.21 ± 0.03 | 0.05 ± 0.02 | < LOD | < LOD | < LOD |
| 364 | 0.22 ± 0.07 | < LOD | < LOD | < LOD | < LOD |
| LOD = Limit of Detection of dimethomorph was 0.040 µg g^-1^ | | | | | |

Table S8. Experimental concentrations of the fungicides dimethomorph and pyrimethanil in T-2 for validating the empirical model

| Fungicide | Day, d | Soil layer depth, cm | | | | |
| --- | --- | --- | --- | --- | --- | --- |
|  |  | **0-20** | **20-40** | **40-60** | **60-80** | **80-100** |
| Dimethomorph | 0 | 0.42 ± 0.10 | < LOD*^a^* | < LOD*^a^* | < LOD*^a^* | < LOD*^a^* |
|  | 1 | 25.38 ± 3.26 | 0.44 ± 0.11 | < LOD*^a^* | < LOD*^a^* | < LOD*^a^* |
|  | 3 | 29.46 ± 2.81 | 0.26 ± 0.07 | < LOD*^a^* | 0.17 ± 0.06 | < LOD*^a^* |
|  | 9 | 10.76 ± 2.53 | 0.18 ± 0.09 | 0.28 ± 0.10 | 0.35 ± 0.09 | 0.48 ± 0.08 |
|  | 16 | 12.84 ± 2.93 | 0.37 ± 0.16 | < LOD*^a^* | 0.24 ± 0.16 | 0.33 ± 0.08 |
|  | 30 | 5.65 ± 0.15 | 0.20 ± 0.07 | < LOD*^a^* | 0.14 ± 0.04 | < LOD*^a^* |
|  | 44 | 6.28 ± 0.72 | 0.17 ± 0.07 | < LOD*^a^* | < LOD*^a^* | < LOD*^a^* |
|  | 58 | 4.40 ± 0.74 | 0.04 ± 0.02 | < LOD*^a^* | < LOD*^a^* | < LOD*^a^* |
|  | 93 | 1.95 ± 0.10 | 0.19 ± 0.09 | < LOD*^a^* | < LOD*^a^* | < LOD*^a^* |
|  | 143 | 2.38 ± 1.32 | < LOD*^a^* | < LOD*^a^* | < LOD*^a^* | < LOD*^a^* |
|  | 255 | 1.38 ± 0.30 | < LOD*^a^* | < LOD*^a^* | < LOD*^a^* | < LOD*^a^* |
|  | 394 | 0.28 ± 0.02 | < LOD*^a^* | < LOD*^a^* | < LOD*^a^* | < LOD*^a^* |
| Pyrimethanil | 0 | 0.83 ± 0.25 | < LOD*^b^* | < LOD*^b^* | 0.14 ± 0.03 | < LOD*^b^* |
|  | 1 | 45.53 ± 5.24 | 0.62 ± 0.11 | < LOD*^b^* | 0.18 ± 0.04 | < LOD*^b^* |
|  | 3 | 48.52 ± 3.10 | 0.35 ± 0.05 | 0.13 ± 0.03 | 0.37 ± 0.08 | 0.11 ± 0.03 |
|  | 9 | 14.47 ± 2.68 | 0.29 ± 0.09 | 0.50 ± 0.13 | 0.65 ± 0.16 | 0.99 ± 0.22 |
|  | 16 | 12.43 ± 1.78 | 0.67 ± 0.26 | < LOD*^b^* | 0.43 ± 0.22 | 0.57 ± 0.15 |
|  | 30 | 4.49 ± 0.96 | 0.25 ± 0.09 | < LOD*^b^* | 0.25 ± 0.02 | < LOD*^b^* |
|  | 44 | 7.04 ± 1.43 | 0.17 ± 0.08 | < LOD*^b^* | < LOD*^b^* | < LOD*^b^* |
|  | 58 | 4.89 ± 0.74 | 0.04 ± 0.01 | < LOD*^b^* | < LOD*^b^* | < LOD*^b^* |
|  | 93 | 2.41 ± 0.28 | 0.30 ± 0.11 | < LOD*^b^* | < LOD*^b^* | < LOD*^b^* |
|  | 143 | 2.87 ± 1.23 | < LOD*^b^* | < LOD*^b^* | < LOD*^b^* | < LOD*^b^* |
|  | 255 | 1.01 ± 0.06 | < LOD*^b^* | 0.11 ± 0.05 | < LOD*^b^* | < LOD*^b^* |
|  | 394 | 0.30 ± 0.08 | 0.17 ± 0.10 | < LOD*^b^* | < LOD*^b^* | < LOD*^b^* |
| *^a^* LOD = Limit of Detection of dimethomorph equal to 0.040 µg g^-1^; *^b^* LOD = Limit of Detection of pyrimethanil equal to 0.036 µg g^-1^ | | | | | | |

**11. Preferential flow**

Fig. S6 shows evidence of preferential flow ways in the greenhouse study plot (area 6587 m^2^). Preferential flow allows rapid movement of water and pollutants through porous soil.

**
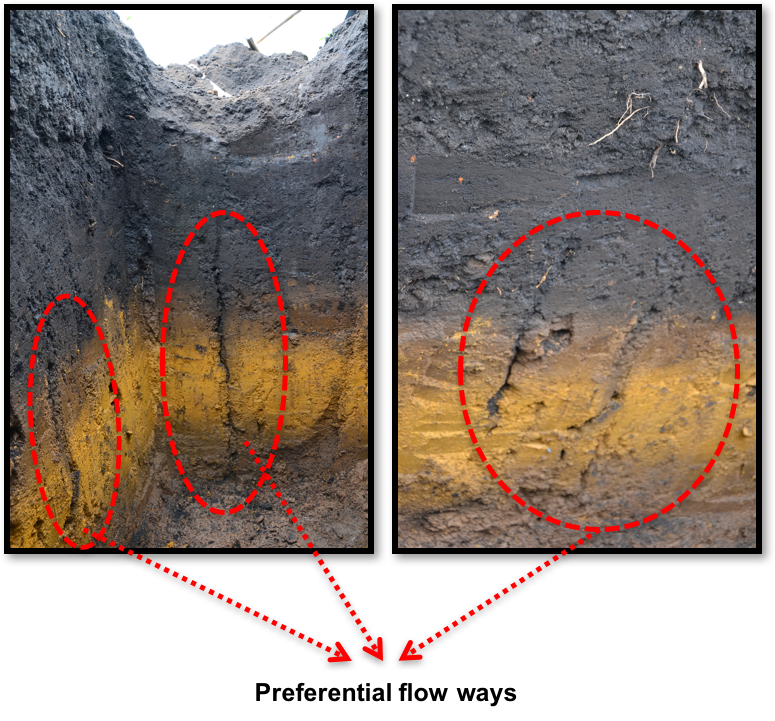
**

Fig. S6. Preferential flow ways in the study plot

**11. Adsorption, single-point desorption, and degradation data.**

Figures S7 and S8 show the observed and estimated (linear model) concentration data generated from adsorption and single-point desorption experiments of pyrimethanil and dimethomorph, respectively. Figures S9 and S10 summarize the observed and estimated (first-order kinetic model) concentration data generated from the degradation of pyrimethanil and dimethomorph, respectively.

Figure S7. Adsorption and single-point desorption isotherms of pyrimethanil for the Andisol soil profile. SL = soil layer, and linear adsorption and desorption lines are the estimated values.

Figure S8. Adsorption and single-point desorption isotherms of dimethomorph for the Andisol soil profile. SL = soil layer, and linear adsorption and desorption lines are the estimated values.

Figure S9. Degradation data of pyrimethanil for the Andisol soil profile. SL = soil layer. The Expon. (Degradation) shows the estimated values based on the first-order kinetic model.

Figure S10. Degradation data of dimethomorph for the Andisol soil profile. SL = soil layer. The Expon. (Degradation) shows the estimated values based on the first-order kinetic model.

**References**

García-Santos G, Scheiben D, Binder CR (2011) The weight method: A new screening method for estimating pesticide deposition from knapsack sprayers in developing countries. Chemosphere 82 (11): 1571-1577.

Goss KU, Scwarzenbach RP (1999) Empirical prediction of heats of vaporization and heats of adsorption of organic compounds. Environ Sci Technol 33(19):3390-3393.

Huysmans M, Dassargues A (2004) Review of the use of Péclet numbers to determine the relative importance of advection and diffusion in low permeability environments. Hydrogeol J 13: 895-904. DOI: 10.1007/s10040-004-0387-4.

Mosquera-Vivas CS, Hansen EW, García-Santos G, Obregón-Neira N, Celis-Ossa RE, González-Murillo CA, Juraske R, Hellweg S, Guerrero-Dallos JA (2016a) The effect of the soil properties on adsorption, single-point desorption, and degradation of chlorpyrifos in two agricultural soil profiles from Colombia. Soil Sci 182:(9-10), 446-456.

Mosquera-Vivas CS, Obregón-Neira N, Celis-Ossa RE, Guerrero-Dallos J A, González-Murillo CA (2016b) Degradation and thermodynamic adsorption process of carbofuran and oxadicyl in a Colombian agricultural soil profile. Agron Colomb 34 (1):92-100.

Mosquera-Vivas CS, Martínez-Cordón MJ, García-Santos G, Guerrero-Dallos JA (2017) Temporal distribution of pyrimethanil and dimethomorph fungicides on an Andisol under cut rose production in Colombia. Höhere Bundeslehr-und Forschungsanstalt für Landwirtschaft Raumberg-Gumpenstein. 17. Gumpensteiner Lysimetertagung 2017, 43 – 48.

Šimůnek J, van Genuchten MTh, Sejna M, Toride N, Leij FJ (1999) Evaluating solute transport in porous media using analytical solutions of the convection-dispersion equation, USDA-ARS U.S. Salinity Laboratory, Riverside, California.

Rao PSC, Hornsby AG, Jessup RE (1985) Indices for ranking the potential for pesticide contamination of groundwater. Proc Soil Crop Sci Soc Fla. 44:1–8.

Vighi M, Di Guardo A (1995) Predictive approaches for the evaluation of pesticide exposure. In: Vighi M. and E Funari (Eds), Pesticide Risk in Groundwater. CRC Press Inc., Boca Raton, FL, pp. 73–100.
